# Characterization of recombinant β subunit of human MUC4 mucin (rMUC4β)

**DOI:** 10.1038/s41598-021-02860-5

**Published:** 2021-12-09

**Authors:** Prakash G. Kshirsagar, Mansi Gulati, Wade M. Junker, Abhijit Aithal, Gaelle Spagnol, Srustidhar Das, Kavita Mallya, Shailendra K. Gautam, Sushil Kumar, Paul Sorgen, Krishan K. Pandey, Surinder K. Batra, Maneesh Jain

**Affiliations:** 1 Department of Biochemistry and Molecular Biology, College of Medicine, University of Nebraska Medical Center, 985870 Nebraska Medical Center, Omaha, NE 68198–5870 USA; 2 Sanguine Diagnostics and Therapeutics, Omaha, NE USA; 3 Department of Molecular Microbiology and Immunology, Saint Louis University Health Sciences Center, St. Louis, MO USA; 4 Fred and Pamela Buffett Cancer Center, University of Nebraska Medical Center, Omaha, NE USA; 5 Eppley Institute for Research in Cancer and Allied Diseases, University of Nebraska Medical Center, Omaha, NE USA

**Keywords:** Biochemistry, Biological techniques, Cancer, Molecular biology

## Abstract

MUC4 is a transmembrane mucin expressed on various epithelial surfaces, including respiratory and gastrointestinal tracts, and helps in their lubrication and protection. MUC4 is also aberrantly overexpressed in various epithelial malignancies and functionally contributes to cancer development and progression. MUC4 is putatively cleaved at the GDPH site into a mucin-like α-subunit and a membrane-tethered growth factor-like β-subunit. Due to the presence of several functional domains, the characterization of MUC4β is critical for understanding MUC4 biology. We developed a method to produce and purify multi-milligram amounts of recombinant MUC4β (rMUC4β). Purified rMUC4β was characterized by Far-UV CD and I-TASSER-based protein structure prediction analyses, and its ability to interact with cellular proteins was determined by the affinity pull-down assay. Two of the three EGF-like domains exhibited typical β-fold, while the third EGF-like domain and vWD domain were predominantly random coils. We observed that rMUC4β physically interacts with Ezrin and EGFR family members. Overall, this study describes an efficient and simple strategy for the purification of biologically-active rMUC4β that can serve as a valuable reagent for a variety of biochemical and functional studies to elucidate MUC4 function and generating domain-specific antibodies and vaccines for cancer immunotherapy.

## Introduction

Mucins comprise a family of 21 high molecular weight secretory or membrane-bound glycoproteins that provide the structural framework of a ‘mucus-like’ layer covering the epithelial surfaces within our body. Their main roles are to protect epithelial surfaces from various insults and promote their regeneration and repair^1–4^. One of the mucins, MUC4, is a large, multi-domain, transmembrane glycoprotein involved in multiple physiological and pathological processes^5^. Full-length MUC4 is synthesized as a single polypeptide that is putatively cleaved in an auto-catalytic manner at the Gly-Asp-Pro-His (GDPH) site located within the vWD domain resulting in two subunits, MUC4α and MUC4β^5–7^. The large extracellular N-terminal subunit MUC4α contains a characteristic tandem repeat (TR) domain along with NIDO and AMOP domains. The smaller, membrane-tethered, C-terminal subunit MUC4β includes a truncated vWD domain, three (EGF)-like domains, and a cytoplasmic tail^8,9^. Due to its aberrant overexpression in multiple carcinomas (pancreas, lung, cervix, ovary, colon, skin), MUC4 has emerged as a promising biomarker and therapeutic target^10–13^. Furthermore, MUC4 is also a potential target for cancer immunotherapy, owing to its aberrant glycosylation and the existence of several splice variants^12,14^. The large, heavily glycosylated MUC4α subunit plays a dominant role in modulating the adhesive properties of epithelial cells^15^. However, the MUC4β subunit harboring three EGF-like domains mediates interactions between MUC4 and EGFR-family proteins (EGFR-1/HER-2/HER-3)^16–20^. These interactions potentiate a diverse array of signaling pathways that result in aggressive growth and enhanced chemoresistance of cancer cells^6,15,16,18^.

We have previously cloned MUC4 “minigene” containing all the MUC4 domains but only 10% of the TR domain and used it to discern the functional role of MUC4 in cancer cell lines using overexpression studies^8,21^. However, direct structure–function studies of MUC4 have been hampered by its large molecular size, lack of domain-specific reagents, and complexities associated with the existence of diverse glycoforms and splice variants in cancer. Purification of the mega-Dalton-sized, ‘full-length’ MUC4 by presently available molecular biology techniques is extremely challenging. Due to its potential retention on the cell membrane post-cleavage, and the presence of several functional domains, we directed our efforts to clone, produce, and purify a recombinant MUC4β (rMUC4β) subunit using a bacterial expression system. Herein, we report the optimization of the parameters for the production and purification of rMUC4β and its preliminary biophysical characterization. ﻿The purified rMUC4β was recently evaluated for vaccine development^22,23^ and the generation of domain-specific monoclonal antibodies (mAbs)^24,25^. We also tested the utility of purified rMUC4β for identifying MUC4-interacting partners. The optimized production and purification method yielded multi-milligram amounts of pure and soluble rMUC4β. In affinity pull-down assays, rMUC4β recognized known (EGFR, HER2, and HER3) and novel (Ezrin) MUC4-interacting partners. Our studies suggest that rMUC4β produced and purified using the optimized method described herein, retains the characteristics of native MUC4, and can serve as a valuable tool to identify MUC4-interacting partners and undertake structure–function studies.

## Results

### Cloning and expression of rMUC4β

**Figure 1:**
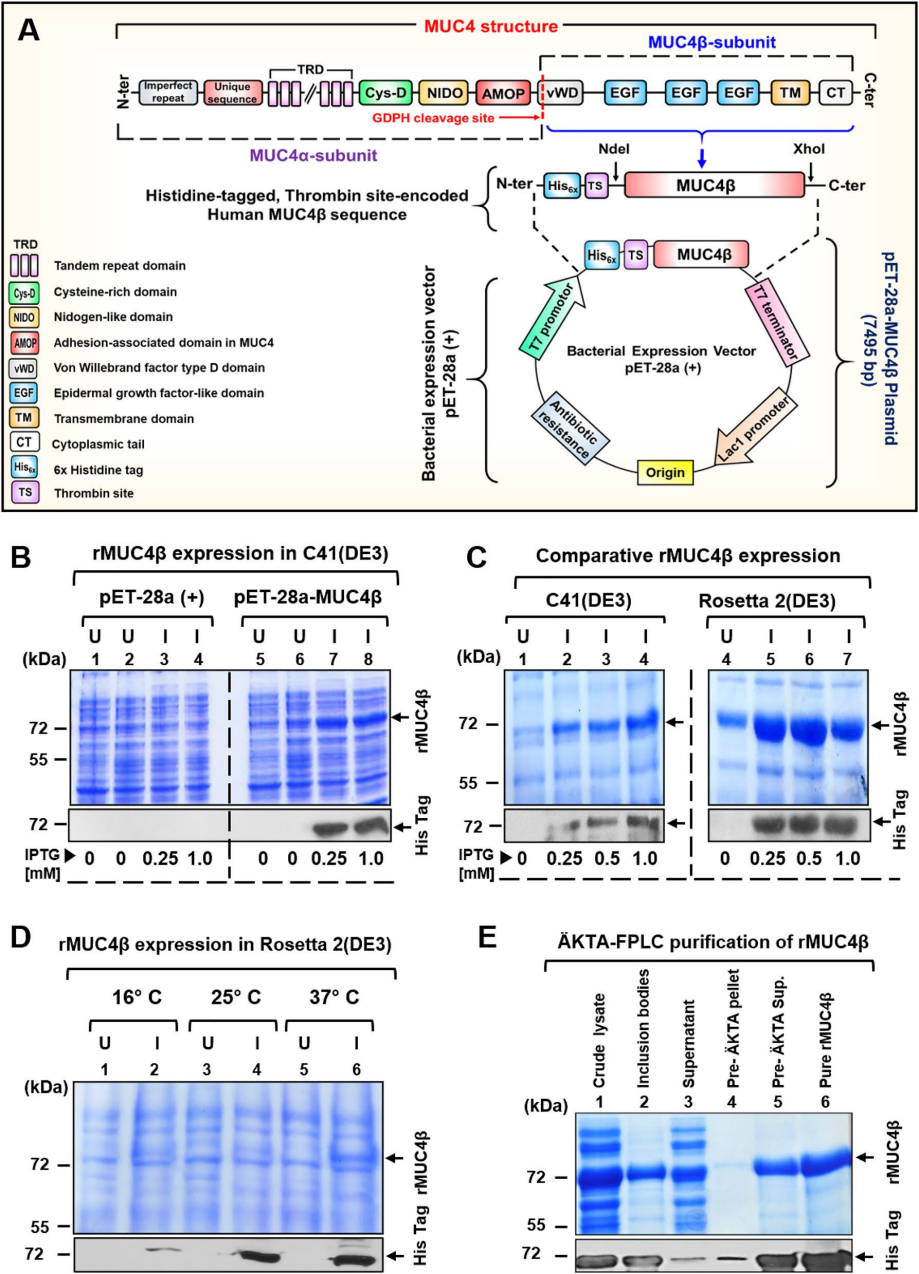
Plasmid cloning strategy, expression, and purification of the human rMUC4β. (**A**) Schematic representation of the complete structure of MUC4 protein and construction of pET-28a-MUC4β plasmid: MUC4β sequence (from GDPH to C-terminal) was amplified with primers incorporated with ‘NdeI’ and XhoI’ restriction sites and cloned in-frame with the His_6x_ tag and thrombin site (TS) encoded by pET-28a (+) vector. The figure was drawn by using Microsoft PowerPoint. (**B**) Expression profile of total cellular proteins from E. *coli* C41(DE3) strain: The pET-28a (empty vector) and pET-28a-MUC4β transformed C41(DE3) cells were induced with an indicated amount of IPTG. SDS-PAGE and immunoblotting analyses were performed on the uninduced (U) and IPTG-induced (I) culture supernatant. (**C**) Comparative assessment of rMUC4β expression profile in C41(DE3) and Rosetta 2(DE3) competent cells at different IPTG concentrations using SDS-PAGE and immunoblotting with anti-His tag antibody. (**D**) Effect of different post-induction incubation temperatures on rMUC4β expression in Rosetta 2(DE3): Total protein expression from each temperature condition was assessed by SDS-PAGE and immunoblotting. (**E**) Isolation and ÄKTA-FPLC affinity purification of rMUC4β. The quality of pre-and-post-ÄKTA fractions was assessed by Coomassie staining and immunoblot analyses.

The schematic structure of MUC4 and the overall scheme of cloning strategy for the construction of the pET28a-MUC4β expression plasmid is described in Fig. 1A. The final construct was sequenced to confirm in-frame insertion of MUC4β with N-terminal hexahistidine (His_6x_) tag (Fig. S1). The overall scheme for the expression and purification of rMUC4β is described in Fig. S2A. Initially, C41(DE3) cells transformed with pET28a-MUC4β were grown until the mid-log phase (Fig. S2B) and induced by the addition of IPTG. The SDS-PAGE and immunoblot analyses of culture supernatants indicated a distinct ~ 72 kDa rMUC4β band following IPTG induction (Fig. 1B, lane 7 and 8), whereas negative control lanes did not show any expression (Fig. 1B, lanes 1–6). The initial purification yield of rMUC4β from a 4 L culture of C41(DE3) cells was found to be low (~ 2–3 mg/L). Hence, we optimized various growth and purification conditions (Fig. S2C-D) to improve the yield and purity of rMUC4β protein.

### Optimization of culture parameters for rMUC4β expression

To improve the yield of purified rMUC4β, we optimized various parameters and examined their effect on protein expression. Competent cells of five different *E. coli* (DE3) strains, namely C41, BL21, C41pLysS, C43pLysS, and Rosetta 2, were transformed and tested for rMUC4β expression. Of all the strains tested, Rosetta 2(DE3) exhibited the highest rMUC4β expression, followed by C41(DE3), while the expression was very low in other strains (Fig. S3A-B). Comparative assessment of rMUC4β expression at variable IPTG concentrations showed higher rMUC4β expression in Rosetta 2(DE3) as compared to C41(DE3) (Fig. 1C). We also examined the effect of different culture media (i.e., Terrific Broth (TB) and LB) on the expression of rMUC4β in Rosetta 2(DE3) at different IPTG concentrations. We observed a ~ 1.3-fold higher expression of rMUC4β in the TB compared to LB (data not shown). However, the cost/benefit provided by the TB media was not substantial enough to justify its use, and thus LB was employed for all further studies. A distinctly higher expression of rMUC4β was observed at 25 °C and 37 °C following IPTG induction, while a relatively low expression was seen at 16 °C (Fig. 1D). To minimize nucleic acid contamination, the Benzonase nuclease treatment was incorporated in the purification scheme (Fig. S2A). Treatment of Rosetta 2(DE3) lysates with Benzonase nuclease for 30 min at 25 °C reduced the viscosity in a dose-dependent manner (Fig. S3C) and hydrolysis of nucleic acids into smaller fragments (< 100 bp) (Fig. S3D, lane 3)^26,27^. Nucleic acids were undetectable in the purified rMUC4β fraction recovered from Benzonase nuclease treated lysate (Fig. S3D, lane 10) compared to untreated lysate (Fig. S3D, lane 11).

### Scale-up production and purification of rMUC4β

Using the optimized conditions (i.e., Rosetta 2 strain, 0.5 mM IPTG, 37 °C, 4 h) for growth and induction, we scaled up the production to obtain a multi-milligram amount of rMUC4β. A major fraction of rMUC4β was observed in the inclusion bodies and was relatively pure (Fig. 1E, Lane 2). Thus, inclusion bodies were isolated by centrifugation, solubilized in 6 M urea, and subjected to purification as outlined in Fig. S2A. The eluted protein was soluble in the 6 M urea but exhibited significant precipitation when urea was removed following dialysis (Fig. S2D). To enhance the protein solubility, a zwitterionic detergent CHAPS was used instead of Triton X-100 in both wash and elution buffers during purification. This led to increased solubility of rMUC4β during the refolding steps and minimized the residual detergent mass in the final lyophilized protein. Using the optimized expression and purification conditions, we accomplished a ~ ninefold increase (i.e., ~ 18 mg/L, + 50% recovery) in the total yield of purified-refolded rMUC4β (Table 1). The purified rMUC4β was observed as a single 72 kDa band in SDS-PAGE (Fig. 1E. Lane 6). Immunoblot analysis of purified protein was also performed for various fractions and conditions to validate using anti-His tag antibody (Fig. 1B–D). Interestingly, immunoblot analysis of the purified protein following BN-PAGE indicated the tendency of rMUC4β to form discrete covalent dimer a ~ 150 kDa under non-reducing conditions, which was resolved into a monomeric form in the presence of BME (Fig. S3E).

**Table 1:** Purification of rMUC4β from *E. coli* Rosetta 2(DE3).

Purification fraction^a^	Total Protein (mg)^b^	rMUC4β (mg)^c^	Purity (%)^d^	Recovery (%)^e^
Crude cellular lysate	313^f^	75	32	100
Inclusion bodies	96	47	41	66
Post-ÄKTA fraction (Non-dialyzed)	48	32	90	70
Purified rMUC4β	22	18	95	55

^a^Fractions collected during ÄKTA-FPLC.

^b^Total protein quantified using NanoDrop One-C (Thermo Scientific).

^c^rMUC4β concentration (mg) determined by the relative quantification of Coomassie Blue stained SDS-PAGE gels compared to a BSA standard curve using Biorad Gel Doc XR + Imaging System (BioRad).

^d^Purity of refolded rMUC4β in each fraction obtained by dividing the rMUC4β band area (intensity) by total area (intensity) of all stained bands in the same lane.

^e^Percent recovery or yield of rMUC4β was calculated on a “per fraction” basis by dividing the amount of MUC4β (mg) in that fraction by the amount present in the previous fraction and multiplying by 100.

^f^Total protein from cell lysate includes both soluble and insoluble protein.

### Prediction of rMUC4β secondary structure by CD spectroscopy

**Figure 2:**
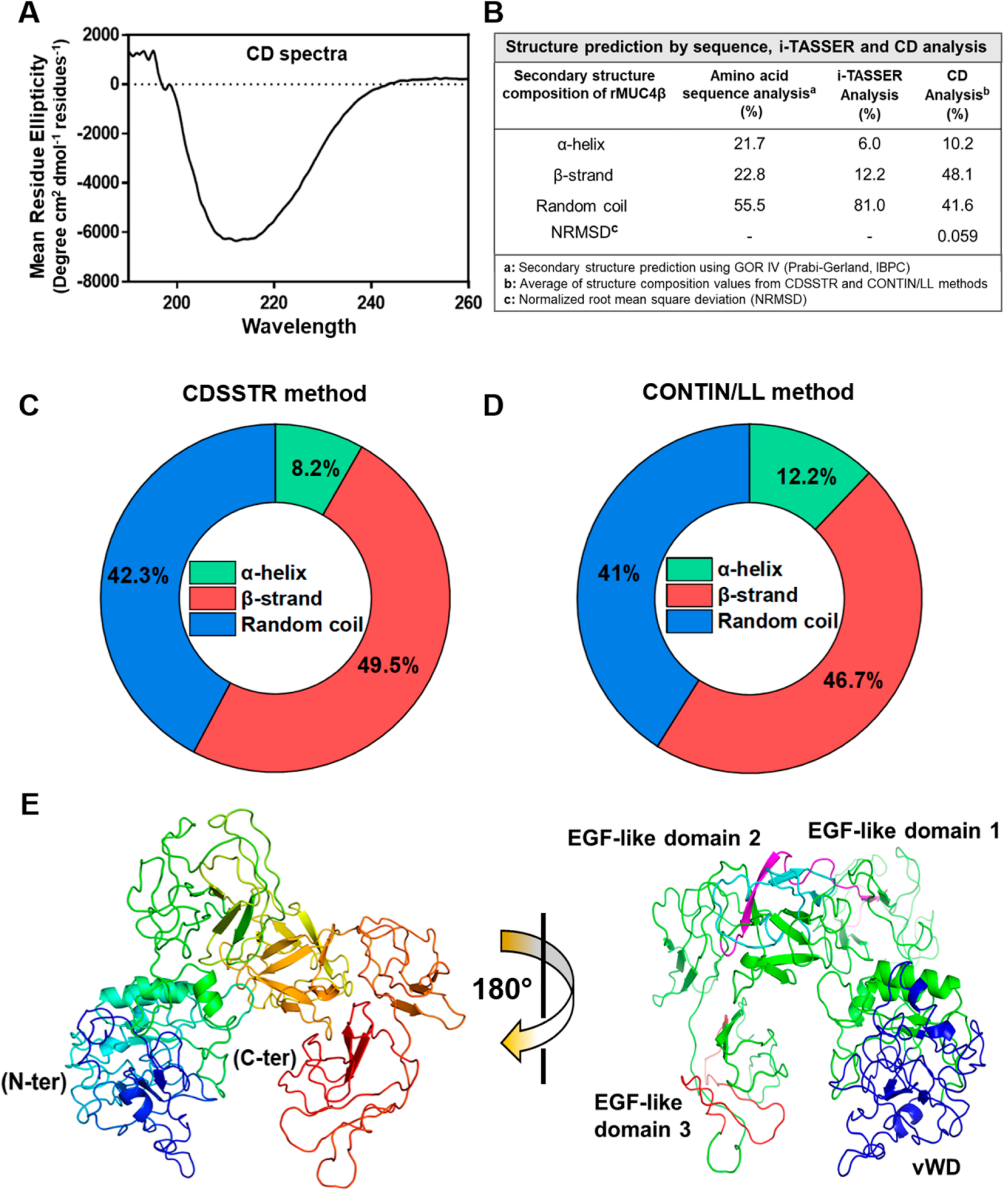
Biophysical characterization and secondary structure analysis of rMUC4β. (**A**) Far-UV CD spectra of rMUC4β were plotted using GraphPad Prism (https://www.graphpad.com/ version 8). (**B**) Comparison of the percentage of secondary structure content estimated or predicted from CD spectrum, amino acid sequence, and I-TASSER analyses. The 2D multicolor pie chart is drawn using Origin 2019 software (https://www.originlab.com/ version 9.6), showing the structure compositions estimated using (**C**) CDSSTR and (**D**) Contin-LL (Provencher and Glockner) methods. (**E**) Predicted structure of rMUC4β generated with the I-TASSER tool (https://zhanggroup.org/I-TASSER/) and PyMOL Molecular Graphics System (https://pymol.org/2/ version 2.3.4), Schrödinger, LLC. The cartoon structure of MUC4β indicating N-terminus (blue) and C-terminus (red). Right panel: i-TASSER predicted structure of rMUC4β rotated 180° to visualize the three EGF-like domains. EGF- like domain I (Cyan) and EGF- like domain II (Magenta) appear to be typical β-strand structures, while EGF-like domain III (Red) is a loop.

To evaluate the stability, folding, and structural integrity of the renatured rMUC4β protein, we performed CD analysis and i-TASSER-based structure prediction. The Far-UV spectrum collected at 7 °C and higher temperatures did not show significant differences (Fig. 2A, S3F). Analysis of the secondary structure recorded at 7 °C is presented in Fig. 2B. The CD spectrum (λ = 190–260 nm) of the refolded rMUC4β (Fig. 2A) is characterized by one broad minima around 215 nm typical of β-strand structure and a lower than expected maxima at about 195 nm that is most likely caused by a significant amount of random coil content (typically shows as a pronounced minima at 197 nm)^28^. Further, the assessment of the CD spectrum (Fig. 2B–D) was performed separately using the CDSSTR and Contin/LL (Provencher & Glockner Method) programs provided by the online analysis tool DichroWeb^29,30^. Both analyses revealed an average secondary structure composition of ~ 10% for α-helical structure, ~ 48% for β-strand, and ~ 41% for random coil (Fig. 2C,D). Comparison of the secondary structure prediction data from the amino acid sequence (GOR IV), I-TASSER, and CD (average composition from the CDSSTR or CONTIN/LL) analyses are shown in Fig. 2B.

### 3-D structure prediction of MUC4β by I-TASSER

To predict the tertiary structure of MUC4β, the amino acid sequence was submitted for 3-D structure prediction to an online server i-TASSER^31–33^. The server-generated five different models were analyzed for the composition of the secondary structure. The percentage of α-helical structures in the 5 predicted models ranged between 1.7 and 6%, while extended β-strands ranged from 10.1 to 29.4%, and random coil structure ranged from 68 to 84% (PDB code: 5G56). Model 1 was chosen for further analysis as it has the highest confidence score (Fig. 2E). The remaining predicted structures were arranged according to their decreasing confidence scores (Fig. S4B-E). The tertiary structure of MUC4β (Model 1) is displayed in a rainbow-colored spectrum (Fig. 2E, left panel), with the N-terminus and C- terminus denoted in blue and red color, respectively. To better visualize the EGF-like domains contained in MUC4β, the structure was rotated 180 degrees. The model revealed that the EGF-like domain 1 (Cyan) and EGF-like domain 2 (Magenta) are composed of the expected typical β folds (anti-parallel β-sheet), while the EGF-like domain 3 (red) is depicted as a mostly random coil structure (Fig. 2E, right panel). Meanwhile, the vWD domain (Blue) appears as a highly random coiled structure; three small α-helix and three β-strands forming a parallel β-sheet) are observed.

### Affinity pull-down and mass spectrometry to detect MUC4 interacting partners

**Figure 3:**
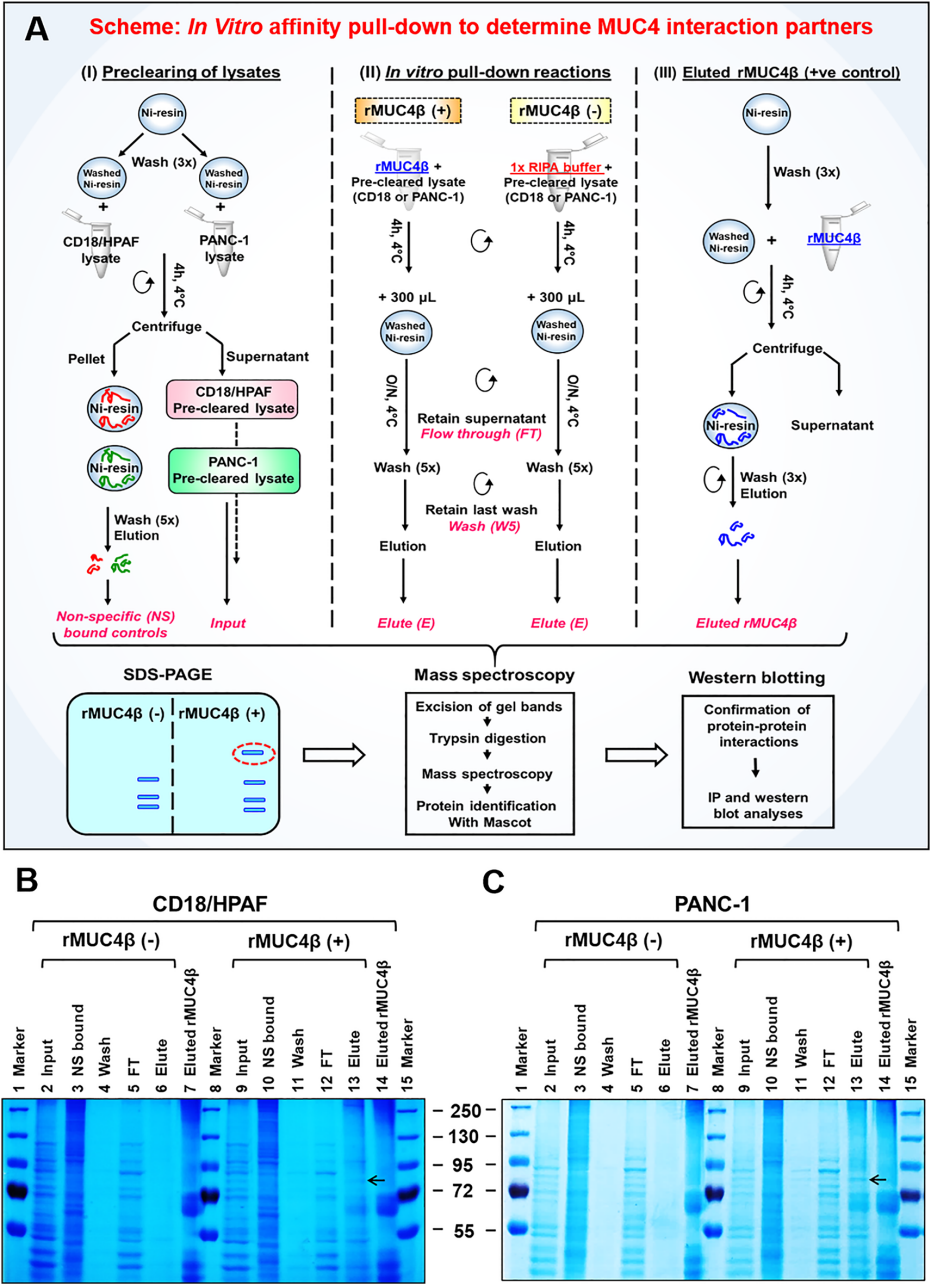
Affinity pull-down and tandem mass spectrometry to determine the interacting partners of MUC4. (**A**) Schematic outline of affinity pull-down assays (I) Pre-clearing of total lysate [input] of MUC4 expressing (CD18/HPAF) and non-expressing (PANC-1) pancreatic cancer cell lines. (II) Affinity pull-down reactions were performed in the presence [rMUC4β (+)] or absence [rMUC4β (−), i.e., RIPA buffer] of rMUC4β (bait) added with washed Ni–NTA beads. Respective supernatants [i.e., Flow-through (FT) and last wash (W5)] were collected before the final elution. (III) An aliquot of rMUC4β-bound Ni-resin was eluted separately to serve as a positive control. All saved fractions (as shown in pink color, italic font) from CD18/HPAF (**B**) and PANC1 (**C**) were resolved on SDS-PAGE gel and stained with Coomassie Blue. The ~ 80 kDa band (arrowed) was identified, excised, and assessed by the tandem MS/MS and proteomic analyses.

**Figure 4:**
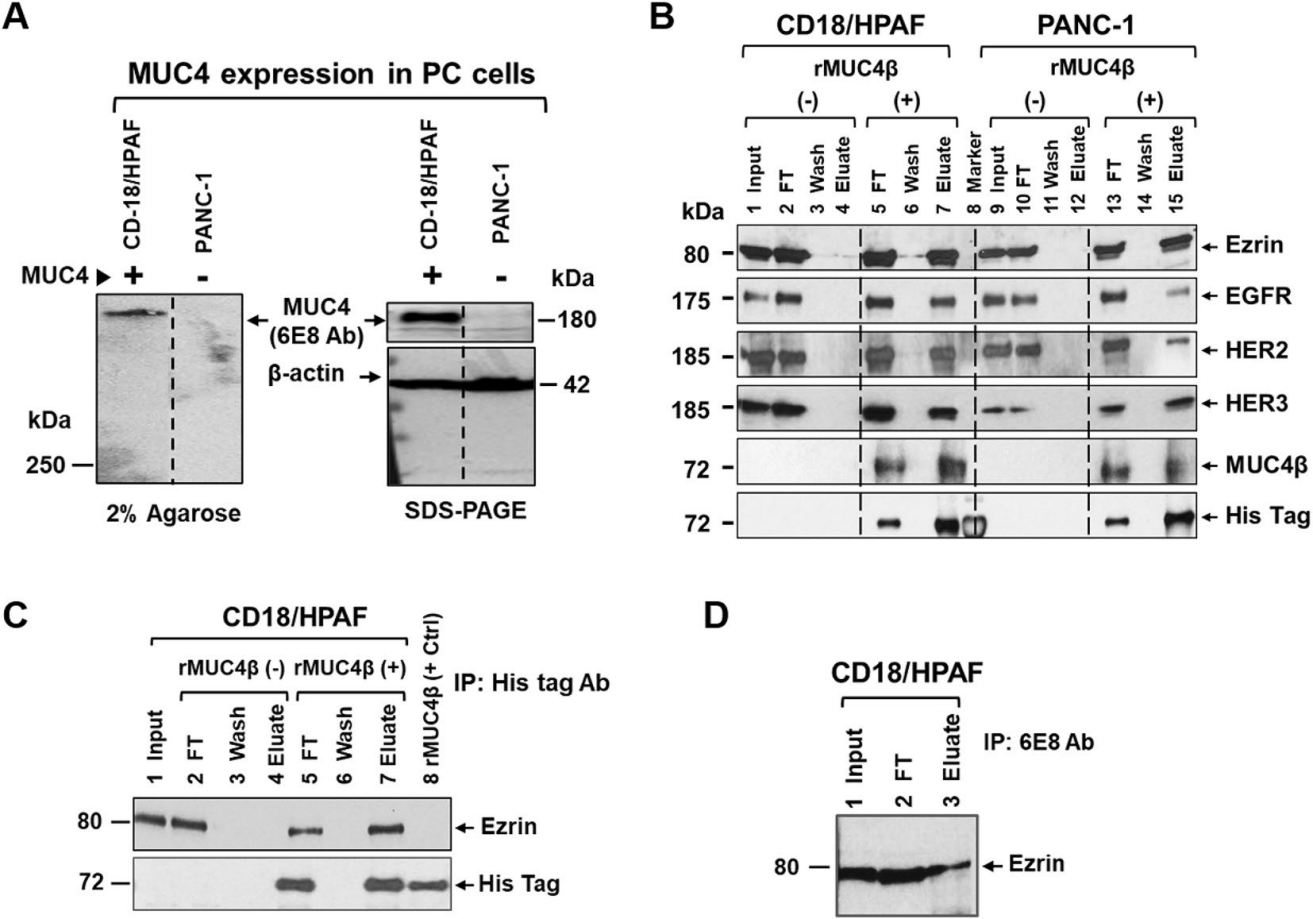
Detection and confirmation of the new and known interacting partners of MUC4 via immunoblotting and IP analyses. (**A**) Expression profile of MUC4 in CD18/HPAF and PANC-1 cells determined by immunoblotting following 2% agarose gel electrophoresis and 10% SDS-PAGE. (**B**) Immunoblot analyses of Ni-NTA pull-down fractions from CD18/HPAF or PANC-1 cell lysates in the presence (+) or absence (−) of rMUC4β. The blots were probed with the indicated antibodies. Input, flow-through (FT), wash, and eluate are the same as described in Fig. 3A. (**C**) Immunoprecipitation using an anti-His tag Ab (clone 27E8) conjugated to magnetic beads following incubation of CD18/HPAF cell lysate in the presence and absence of rMUC4β. (**D**) Interaction of endogenous MUC4 with Ezrin. Immunoprecipitation was performed with protein A/G agarose beads on CD18/HPAF lysates following incubation with anti-MUC4β Ab.

To determine if the rMUC4β exhibits a similar conformation to native MUC4, we tested its ability to interact with known and potentially novel MUC4-interacting partners using a His-affinity pull-down assay as outlined in Fig. 3A. We observed that rMUC4β pulled down a ~ 80 kDa band from the lysates of both MUC4-expressing (CD18/HPAF) and non-expressing (PANC-1) cell lines (Fig. 3B,C, lane 13), which was not detected in the pull-down eluates when beads alone were used (Fig. 3B,C, lane 6). This band was excised, in-gel trypsin digested, and the resulting peptides were analyzed by tandem MS/MS. The most significant top hits were matched to Ezrin in CD18/HPAF and PANC-1 cells (Fig. S5A-B). Analysis of the pulled-down complexes by immunoblotting with the antibodies of known MUC4-interacting proteins (EGFR family receptors) indicated that rMUC4β interacts with EGFR, HER2, and HER3 in both MUC4-expressing and non-expressing cells (Fig. 4A,B). Interaction with Ezrin was confirmed by a pull-down assay using an anti-His tag Ab-coupled to magnetic beads to immobilized rMUC4β (Fig. 4C). Finally, the ability of endogenous MUC4β to interact with Ezrin was further confirmed by CO-IP (i.e., immunoprecipitation with anti-MUC4β mAb 6E8 (Fig. 4D). Overall, these results suggest that the rMUC4β recapitulates the functional conformation of native MUC4β and can recognize its natural interacting partners.

## Discussion

Due to their differential expression in various malignancies, mucins have been extensively investigated as determinants of disease initiation and progression and as diagnostic and therapeutic targets^1,34–36^. MUC4 is a high molecular weight, multi-domain, and heavily glycosylated protein that is differentially overexpressed in multiple cancers, including pancreatic, breast, lung, and head and neck cancers, where it functionally contributes to disease initiation, progression, metastasis, and chemoresistance^5,9,11,13,37,38^. It is also a potential biomarker for the diagnosis and prognosis of various cancers and a novel target for cancer therapeutics^11–13^.

Most information regarding MUC4 function has been discerned using cell lines engineered for MUC4 overexpression or knockdown, while its pathobiological significance has been determined from correlative studies involving the analysis of biological samples for MUC4 transcripts and protein. However, direct structure–function studies for human MUC4 have not been extensively performed due to its large genomic and molecular size, and extensive glycosylation. Due to the large size of the MUC4 gene and transcript, cloning and expressing a full-length MUC4 protein is practically impossible. We have previously cloned the MUC4 “minigene” in a eukaryotic expression system, containing only 10% of the tandem repeat sequence but with all other domains intact^8,21^. While this construct has been useful for studying the impact of MUC4 overexpression in non-expressing cells to discern MUC4 function, it is unsuitable for large-scale purification. An alternative approach is to clone, express, and purify smaller fragments containing functional domains. We have previously cloned and expressed various domains of the MUC4α subunit and used them developing domain-specific anti-MUC4 monoclonal antibodies^39^. Due to the presence of three EGF-like domains and a vWD domain, and its proximity to the cell membrane, several functions of MUC4, particularly the ones involving interactions with the EGF receptor family^18,19,38^, have been attributed to MUC4β subunit.

Further, our recent analysis suggests that the immunogenic peptides in MUC4 are enriched in the β-subunit^12^. Due to these attributes, the MUC4β subunit is a potential target for immunotherapy and targeted therapies^11,12,29^. Thus, we directed our efforts to clone, express, and purify the MUC4β subunit with a goal to utilize the purified recombinant protein to develop domain-specific antibodies and pancreatic cancer immunotherapy. Recently, Liberelle et al*.* generated fusion constructs containing GFP fused to either intact human MUC4β or truncated mutants containing only EGF-like domains. These constructs were expressed in CHO cells and used to characterize MUC4-ErbB2 interactions. However, the studies were performed using CHO cell lysates and recombinant ErbB2, and no efforts were made to purify MUC4β-GFP^19^.

Initially, the *MUC4β* sequence was cloned into the pET-28a (+) expression vector. Initial scale-up attempts using 1L culture of pET28a-MUC4β plasmid transformed in C41(DE3) cells resulted in a low yield of purified MUC4β (i.e., ~ 1.8 mg/L of LB media). Analysis of MUC4β sequence, with a rare codon calculator (RaCC), indicated the presence of 32 rare or minor codons including AGA, AGG, CGA, CGG, CUA, CCC, and AUA (https://web.expasy.org/protparam/). Rare or minor codons are underutilized codons by the bacterial translational machinery, resulting in low protein yield. We screened five different genetically engineered *E. coli* strains for MUC4β expression, of which Rosetta 2(DE3) provided the maximum improvement (> tenfold than the C41 strain) in protein yield (Table 1). This specific strain expresses the less utilized tRNAs to compensate for rare codons that are generally used in eukaryotes but rarely used in *E. coli*^40–42^. Prior to scaling up the production, we optimized various parameters, including post-induction incubation temperature, the concentration of IPTG, and culture media composition, to improve the rMUC4β yield. Under conditions optimized for high expression (IPTG- 0.5 mM; growth medium-LB broth), rMUC4β accumulated in inclusion bodies. We developed a comprehensive purification strategy that included cell lysis, removal of nucleic acids, isolation and solubilization of inclusion bodies, immobilized metal affinity chromatography, and dialysis to facilitate urea removal and protein refolding. Due to the high amount of nucleic acids, the high viscosity of the bacterial lysates can potentially interfere with downstream processing and impact protein yield. Reduction in cell lysate viscosity can be achieved by supplementing bacterial lysate with Benzonase nuclease^26,28,43^. The use of CHAPS instead of non-ionic Triton X-100 in the solubilization and elution steps prevented protein aggregation during urea removal and contributed to the high solubility of rMUC4β in water that can be attributed to the high CMC value and zwitterionic nature of CHAPS^44^. Immunoblotting analysis following BN-PAGE under reducing and non-reducing conditions suggested the tendency of purified rMUC4β to form covalent dimers via disulfide bonds. The possibility of higher-order soluble aggregates can be ruled out as we did not observe higher molecular weight bands even by immunoblotting.

To gain insight into protein folding, we employed a GOR IV server to predict the secondary structure by amino acid sequences. Further, we performed structural characterization of the purified rMUC4β protein by conducting CD spectroscopy. The CDSSTR and Contin/LL methods were employed to analyze the Far-UV CD spectrum^29,30,45^. CD data demonstrated the presence of α-helix, β-strand, and random coils. While understanding the secondary structure, we observed structural disparity in the β-structure derived from CD and other secondary structure sequence prediction methods. Supportively, some reports have shown some discrepancies in CD analysis and sequence-based structure predictions regarding the quantification for β-structure because of their morphological and structural diversity^46–48^. To gain further insights into the tertiary structure and understand the folding of the rMUC4β, we performed structure prediction analysis using an online-based I-TASSER server.

The server-generated five models. The model with the highest confidence score was selected to understand the folding pattern and its interacting partners. The predicted tertiary structure showed typical β-folds for two EGF-like domains, while the third EGF-like domain showed a random coiled structure. The vWD domain is predominantly disordered, but we did observe a parallel β-sheet comprising of three β-strands (3–4 residues each). Three small α-helical structures (4 residues each) were also observed in the vWD domain. The presence of a high percentage of random coils (disordered structures) in MUC4 can be attributed to the existence of a high proportion of charged or polar residues. The decreased hydrophobic residues make the protein less susceptible to present a hydrophobic ordered core^49,50^. In a recent study analyzing the intrinsically disordered structures of mucins, we reported that MUC4 is 77% disordered; the study also highlighted the vWD domain as a highly disordered domain^51^. However, these prediction studies are based on apomucin backbones, and the impact of glycosylation on the intrinsically disordered content of mucins (including MUC4) remains unexplored and remains to be discerned. While the recombinant MUC4β protein is non-glycosylated, our affinity pull-down studies demonstrated the ability of MUC4β to interact with EGFR and HER2 in a manner similar to that reported previously for native protein^17–19,52^. Thus, some of the interactions of MUC4 with its binding partners can be glycosylation independent. We observed some differences in the predicted structures by GOR IV and i-TASSER; these differences can be attributed to the different algorithms employed by these two servers. GOR IV is a secondary structure prediction method that uses a probability-based algorithm and performs the jack-knife method to determine the secondary structure. This method aligns the target sequence with the GOR database of 267 sequences and has a prediction accuracy of about 64%^53^. The tertiary structure predicted by i-TASSER involves structure prediction by identifying the structure templates by Local Meta-Threading Server (LOMETS) and other threading software, which generate thousands of structure alignments^54^. The structures with the highest significance are ranked based on Z-scores. Both methods utilize different approaches to predict the structure of the target sequence; therefore, we observe differences in the predicted structures. The structural and functional characterization of the rMUC4β paves a path to further explore its potential as a therapeutic agent.

MUC4 plays multifaced roles in cell adhesion, migration, proliferation to promote tumorigenesis and metastasis in pancreatic cancer and other epithelial malignancies^5,6,37,38^. One of the ways by which it mediates these roles is by interacting with cell surface molecules such as HER2, HER3, and integrins and soluble mediators in the extracellular microenvironment such as galectins^16,18,20,55^. To determine whether rMUC4β achieves a folding state and is biologically active, we performed an affinity pull-down assay utilizing rMUC4β as the ‘bait’ to isolate putative interacting partners from RIPA extracted lysates of a MUC4 expressing (CD18/HPAF) and a MUC4 non-expressing (PANC-1) cell line. A ~ 80 kDa band was observed in the eluted fraction that was identified as ‘Ezrin’ by MS/MS analysis. Ezrin is a cytoskeletal protein belonging to the Ezrin-Radixin-Moesin (ERM) family that plays an essential role in cell adhesion, motility, invasion, cancer progression, and metastasis^56–58^. Recently, the TCGA and CCLE datasets were used to identify genes for which expression is correlated with MUC4. EZRIN was one of the 178 reported genes with a Pearson correlation higher than 0.3^17^. However, to the best of our knowledge, no study has yet provided any experimental evidence suggesting the possible physical interaction between MUC4 and Ezrin. Interestingly, it has been shown that the cytoplasmic tail of another mucin, MUC16, interacts with the ERM family of proteins via its characteristic polybasic sequence^59^, connecting it to the actin cytoskeleton in the cytoplasm. It is possible that mucin-ERM interactions might be an important phenomenon and have functional relevance that is yet to be described.

We also confirmed the interaction of known interacting partners of MUC4 by immunoblotting the eluted fraction with specific antibodies. Both HER-2 and HER-3 were shown previously to interact with MUC4^17–19^ and are detected in the eluted fraction following multiple pull-down assays described with rMUC4β. This further confirms that the interaction of these receptors with MUC4 is via β-subunit and is independent of glycosylation. Interestingly, we could also detect EGFR in the eluted fraction. This can either be due to direct interaction with the protein or indirectly because of its association with HER2. Given the folding pattern observed in our structure prediction analysis, it is possible that the three EGF-like domains of MUC4 bind to the EGF binding pocket of EGFR. Further studies are needed to confirm or rule out these observations. It will be interesting to determine if exogenously added rMUC4β can potentially compete with the endogenous MUC4 for binding partners and serve as a potential therapeutic agent.

In summary, we successfully developed an expression and purification system to produce multi-milligram amounts of rMUC4β. The recombinant protein fragment retained the binding characteristics of the native MUC4β and can be a valuable tool to undertake MUC4 structure–function analysis. Further, purified rMUC4β has been successfully formulated and evaluated as a nanovaccine^22,23^ and used as an immunogen for the development of domain-specific anti-MUC4 antibodies^24,25^.

## Materials and methods

### Materials

C41(DE3) and BL21(DE3) *E. coli* strains were purchased from MilliporeSigma. C41pLysS, C43pLysS, and Rosetta 2(DE3) *E. coli* strains were procured from Thermo scientific. Bacto tryptone, Bacto yeast, and Bacto Agar were purchased from BD Biosciences. Anti-His tag Ab (Clone 27E9, Cat. No. 2366) and anti-EGFR mAb (Clone D38B1, Cat. No. 4267) were purchased from Cell Signaling Technology, while anti-Ezrin Ab (Clone H-276, Cat No. SC-20773) was bought from Santa Cruz Biotechnology. HisTrap HP affinity Ni-NTA column was purchased from GE healthcare. Anti-His tag Ab Magnetic bead conjugate (Cat. No. 8811) and Protein A/G agarose beads (Cat. No. 9863) were purchased from Cell Signaling Technology. Anti-MUC4β mAb (Clone 6E8) was generated using recombinant rMUC4β protein as an immunogen. More details on the construction of recombinant pET-28a-MUC4β are mentioned in supporting information.

### Optimization of culture parameters to enhance rMUC4β yield

#### Selection of *E. coli* host for optimal production

Five different chemically competent *E. coli* (DE3) strains (C41, BL21, C41pLysS, C43pLysS, and Rosetta 2) were tested for optimal expression of rMUC4β protein. Briefly, selected strains transformed with pET28a-MUC4β plasmid were grown in LB/antibiotics media overnight at 37 °C. A freshly-made pre-inoculum culture (2%) from each cell type was inoculated into 20 mL LB media and incubated at 37 °C until the mid-exponential growth phase. When OD_600 nm_ reached ~ 0.6, cultures were induced with 0.5 mM IPTG for 4 h at 37 °C (a set of negative control was incubated under similar conditions without IPTG). Next, both induced and uninduced fractions were harvested and disrupted in an appropriate volume of lysis buffer (500 mM Tris HCl, 2 mM MgCl_2_, 10 mM NaCl, pH 8.0) to adjust the OD_600 nm_ to ~ 1.2/mL. Equivalent amounts of crude lysate were loaded, ran on SDS-PAGE, and stained with Coomassie brilliant blue.

#### Optimization of rMUC4β expression

C41(DE3) and Rosetta 2(DE3) competent cells transformed with pET28a-MUC4β plasmid were grown in LB culture at 37 °C. After reaching the mid-exponential phase, all bacterial cultures were separately induced at variable IPTG concentrations (0, 0.25, 0.5, and 1.0 mM) for 4 h at 37 °C. Uninduced controls were prepared without adding IPTG. The effect of three different post-induction temperatures on the enrichment of rMUC4β expression was evaluated in pET-28a-MUC4β transformed Rosetta 2(DE3) cells^60^. Bacterial culture was induced at ~ 0.6 OD by adding 0.5 mM of IPTG. After induction, each tube was incubated separately at 16 °C, 25 °C, and 37 °C, respectively, for 4 h. To investigate the effects of different culture media on the expression efficiency of rMUC4β in *E. coli*, freshly-made pre-inoculum culture (2%) of Rosetta 2(DE3) cells transformed with pET-28a-MUC4β plasmid was added to 20 mL of culture media (LB and TB) and grown at 37 °C until medium exponential phase. One set of cultures was induced with 0.5 mM ITPG and incubated for 4 h at 37 °C. The second control set was grown without IPTG addition. A sample from each culture condition was pelleted and lysed in an appropriate volume of lysis buffer to maintain equal ODs (~ 1.2/mL). After lysis, SDS-PAGE and immunoblotting analysis were performed using equal amounts of lysates to identify IPTG concentration, temperature, and culture media for optimal production.

### Scale-up production of rMUC4β protein in Rosetta 2(DE3)

Large-scale production of rMUC4β protein was undertaken using optimized culture conditions. The transformed cells were grown on LB agar plates containing kanamycin (50 µg/mL) and chloramphenicol (40 µg/mL). After overnight incubation at 37 °C, a single colony was picked and grown overnight into 10 mL of LB/antibiotics culture in a shaking incubator at 37 °C. Following day, 50 mL of LB/antibiotics culture was inoculated by 500 µL of overnight-grown culture (1%) and incubated overnight at 37 °C, 250 rpm. The next day, overnight grown pre-inoculum (15.0 ml) was inoculated into 1 L of LB/antibiotics media. Subsequently, the culture was incubated under continuous agitation at 37 °C until OD_600 nm_ reached ~ 0.5–0.6. MUC4β expression was induced by adding IPTG [0.5 mM] and growing for another 4 h at 37 °C. Cells were harvested by centrifugation (4300 × g, for 30 min, at 4 °C), washed once with ice-cold 1 × PBS, and stored overnight at -80°C^61^. The bacterial pellet was thawed and resuspended in ice-cold lysis buffer [50 mM Tris–HCl (pH 8.0), 2 mM MgCl_2_, and 150 mM NaCl, EDTA-free protease inhibitor cocktail (250 µL/10 g of the bacterial pellet) and benzonase nuclease (~ 10 U/mL pre-lysis culture). Following 15 min incubation on ice, cells were lysed with three passages through an EmulsiFlex-C3 (Avestin) at ~ 15,000 psi. Cell debris was removed by centrifugation (4300 × g for 30 min at 4 °C). The supernatant was treated again with benzonase nuclease (10 U/mL cell lysate) and incubated for 2 h at 4°C^43,62^. The rMUC4β protein present in the insoluble inclusion bodies was collected by high-speed centrifugation (39,000 × g for 60 min at 4 °C). The recovered pellet was resuspended in 40 mL of buffer A (1 × PBS, 6 M urea, 20 mM imidazole, 300 mM NaCl, 0.5% CHAPS, and 2 mM β-mercaptoethanol (BME), pH 8.0), incubated overnight at 4 °C and again treated with Benzonase nuclease. Insoluble debris was removed by centrifugation (39,000 × g, 60 min, at 4 °C), and the pre-ÄKTA supernatant was passed through a 0.22 µm Steriflip vacuum filtration unit (EMD Millipore). More details on the purification and refolding of rMUC4β protein can be found in supporting information.

### Purification and protein refolding

The solubilized inclusion bodies (pre- ÄKTA supernatant) recovered after multi-step centrifugation was filtered and applied to a HisTrap Ni–NTA column (GE Healthcare) connected to an ÄKTA-FPLC (GE Healthcare). The column was washed with buffer A. Protein elution was performed using a step gradient consisting of 20%, 30%, 50%, 60%, 75% and 100% Buffer B (1 × PBS, 6 M urea, 500 mM imidazole, 300 mM NaCl, 0.5% CHAPS, and 2 mM BME, pH 8.0). Eluted fractions were run on SDS-PAGE gels and were visualized by Coomassie staining. Fractions containing His_6x_-tagged MUC4β were pooled and concentrated using Amicon Ultra-4 centrifugal filters (50 kDa MWCO) to exclude lower molecular weight bacterial protein impurities. For protein refolding, a Float-A-lyzer G2 dialysis tube (50 kDa MWCO, Spectrum Labs) was used, and step-wise dialysis was performed against 1 × PBS (pH 8.0, volume 2 L) containing decreasing concentrations of urea (6 M, 5 M, 4 M, 3 M, 2 M, 1 M, and 1 × PBS) to gradually minimize the denaturant concentration and allow for a smooth refolding of rMUC4β^63,64^. Each dialysis step was performed at 4 °C for 3 h against 2 L volumes of dialysis buffer. The final dialysis was done against Hyclone (GE) endotoxin-free water (for 15 h with three changes every 5 h). Purified rMUC4β was also resolved using Blue Native PAGE (BN-PAGE), following thermal denaturation under both reducing and non-reducing conditions, subjected to western blotting and probed with anti-His tag mAb to determine the presence of covalent and/or non-covalent dimers or aggregates^65^.

### Circular dichroism (CD) spectroscopy

Circular dichroism (CD) analysis of rMUC4β was performed on a Jasco J-815 spectrometer in the Far-UV spectral region (λ = 190 to 260 nm) using a 0.1 mm path length quartz cell. CD spectra of rMUC4β at a concentration of 39 μmol/L in 1 × PBS were recorded at 7 °C, 25 °C and 37 °C using a scan rate of 50 nm/min, an integration time of 1 s, and a bandwidth of 1.0 nm. The final spectrum was obtained by averaging five scans and was corrected by subtracting the solvent spectrum acquired under identical conditions. CD data were processed using the spectra analysis function of Jasco Spectra Manager II (https://jascoinc.com/). Analysis of the CD spectrum was performed separately using two methods, CDSSTR and Contin/LL provided by DichroWeb (http://dichroweb.cryst.bbk.ac.uk/)^30^.

### Iterative threading ASSEmbly refinement (I-TASSER)

I-TASSER is a bioinformatics program that predicts the three-dimensional (3-D) model structure of the desired protein using amino acid (AA) sequences. We used this intelligent program to model the 3-D structure of the MUC4β protein by submitting the corresponding AA sequences to the I-TASSER online server^31–33^. After obtaining five different predicted models of MUC4β, the structure with the highest confidence score was selected for comparative analysis with CD data.

### Affinity pull-down assays

High-affinity Ni–NTA resin (GenScript, Cat. No. L00250) was washed and equilibrated with ice-cold wash buffer (1 × PBS, containing 1% CHAPS, 20 mM imidazole, and 2 mM BME, 300 mM NaCl, pH 8.0). The equilibrated Ni–NTA slurry was incubated with either CD18/HPAF or PANC-1 cell lysates for 4 h at 4 °C to pre-clear the cell lysates. Ni–NTA resin, after pre-clearing for each sample, was used as a “non-specific binding control.” A portion of each pre-cleared lysate supernatant was saved and used as ‘Input’ control. The lysates were incubated with 60 μg of rMUC4β protein [rMUC4β (+)] or without rMUC4β protein [rMUC4β (−)], in total 500 µL reaction volume overnight at 4 °C with end-over-end mixing. The samples were centrifuged (2000 rpm, for 2 min at 4 °C), and the supernatant containing unbound proteins was designated as “flow-through” (FT) and saved for further analysis. The Ni-NTA resin was washed five times (2000 rpm, for 2 min at 4 °C) with ice-cold wash buffer (1 × PBS, containing 20 mM imidazole, 1% CHAPS, 2 mM BME, 300 mM NaCl, pH 8.0) and the last wash (Wash 5) was saved for analysis. Finally, the ‘bait-prey’ complexes were eluted with elution buffer (1 × PBS supplemented with 500 mM imidazole, 1% CHAPS, and 2 mM BME, 300 mM NaCl, pH 8.0) ice for 15 min. The eluted proteins (Eluate) were recovered in the supernatant following centrifugation. An aliquot of rMUC4β-bound Ni-resin was also eluted with elution buffer to serve as a positive control (i.e., Eluted Recombinant Protein, “ERP (+)” control). All collected samples were solubilized in 6 × Laemmli sample buffer, heated at 95 °C for 5 min, and subjected to SDS-PAGE and immunoblot analyses briefly described in supporting information.

### Mass spectrometry (MS/MS) characterization of excised protein band

The protein profiles of pull-down eluted complexes in two different cell lysates (CD18/HPAF and PANC-1) were visualized by Coomassie staining and silver staining. The distinct protein band present in the eluted fraction of the rMUC4β (+) panel but absent in the eluted fraction rMUC4β (−) panel in both CD18/HPAF and PANC-1 lysates was destained and stored in 50% methanol overnight at 4 °C. The selected band (MW ~ 80 kDa) was excised and digested with trypsin by the UNMC Mass Spectrometry and Proteomics Core. Following digestion, samples were dried by SpeedVac, resuspended in 20 µl of 0.5% trifluoroacetic acid, and were cleaned up using ZipTip µC18 columns (EMD Millipore). Further, the samples were resuspended in 0.1% formic acid and injected onto an Eksigent cHiPLC column (75 µm × 15 cm ChromXP C18-CL 3 µm 120 Å), and 6600 TripleTOF instruments (gradient 2–60% ACN in 60 min).

## Supplementary Information

Supplementary Information.

## Abbreviations

Ab: Antibody
EGFR: Epidermal growth factor receptor
FPLC: Fast protein liquid chromatography
MW: Molecular weight
MUC4: Mucin 4
MWCO: Molecular weight cut-off
PCR: Polymerase chain reaction
TB: Terrific broth
LB: Luria broth
BME: β-Mercaptoethanol
vWD: Von Willebrand factor type D domain
PIC: Protease inhibitor cocktails
FPLC: Fast pressure liquid chromatography
