# Supplementary material for: Characterization of recombinant β subunit of human MUC4 mucin (rMUC4β)
Source: Sci Rep. 2021 Dec 9;11:23730. doi: 10.1038/s41598-021-02860-5 (PMC8660890; doi:10.1038/s41598-021-02860-5)
Supplement: Supplementary file 1 — Supplementary Information. [file 41598_2021_2860_MOESM1_ESM.docx]

**Supporting Information**

**Characterization of** **Recombinant β subunit of Human MUC4 mucin (rMUC4β)**

Prakash G. Kshirsagar^1^, Mansi Gulati^1*^, Wade M. Junker^1,2*^, Abhijit Aithal^1*^, Gaelle Spagnol^1^, Srustidhar Das^1^, Kavita Mallya^1^, Shailendra K. Gautam, Sushil Kumar^1^, Paul Sorgen^1^, Krishan K. Pandey^3^, Surinder K. Batra^1,2,4,5^, Maneesh Jain^1,4^

^1^Department of Biochemistry and Molecular Biology, University of Nebraska Medical Center, Omaha, Nebraska, USA.

^2^Sanguine Diagnostics and Therapeutics, Omaha, Nebraska, USA.

^3^Department of Molecular Microbiology and Immunology, Saint Louis University Health Sciences Center, St. Louis, Missouri, USA.

^4^Fred and Pamela Buffett Cancer Center, University of Nebraska Medical Center, Omaha, Nebraska, USA.

^5^Eppley Institute for Research in Cancer and Allied Diseases, University of Nebraska Medical Center, Omaha, Nebraska, USA.

**For correspondence:**

Maneesh Jain, Ph.D. Email: [mjain@unmc.edu](mailto:mjain@unmc.edu) Phone: 402-559-7667

Surinder K. Batra, Ph.D. Email: [sbatra@unmc.edu](mailto:sbatra@unmc.edu) Phone: 402-559-5455

Department of Biochemistry and Molecular Biology, College of Medicine, University of Nebraska Medical Center, 985870 Nebraska Medical Center, Omaha, NE 68198–5870.

Phone: 402-559-7667 Fax: 402-559-6650.

***** These authors contributed equally

**Supporting figures**

| **Figure S1.** Recombinant pET-28a-MUC4β vector construct (plasmid), nucleotide, and amino acid sequences of the inserted gene. | **3** |
| --- | --- |
| **Figure S2.** rMUC4β protein isolation and ÄKTA-FPLC purification. | **4** |
| **Figure S3.** Optimization of conditions for rMUC4β expression. | **6** |
| **Figure S4.** Secondary structure prediction of MUC4β by I-TASSER. | **8** |
| **Figure S5.** List of proteins identified through mass spectrometry in the ~80 kDa band and proteomic analysis performed using Mascot. | **10** |
| **FigureS6-S17.** Full-length SDS-PAGE gel or western blot images of figure 1B, 1C, 1D, 1E, 1E (Biological replicate 1), 3B, 3C, 4A, 4B, 4B (Biological replicate 1), 4C, 4D, S3A, S3D and S3E. | **11-27** |

**Supplementary methods**

| **S1.** Construction of recombinant plasmid encoding for MUC4β expression. | **28** |
| --- | --- |
| **S2.** Cell culture and protein lysate preparation. | **28** |
| **S3.** SDS-PAGE and immunoblot analysis. | **28** |
| **S4.** References. | **29** |


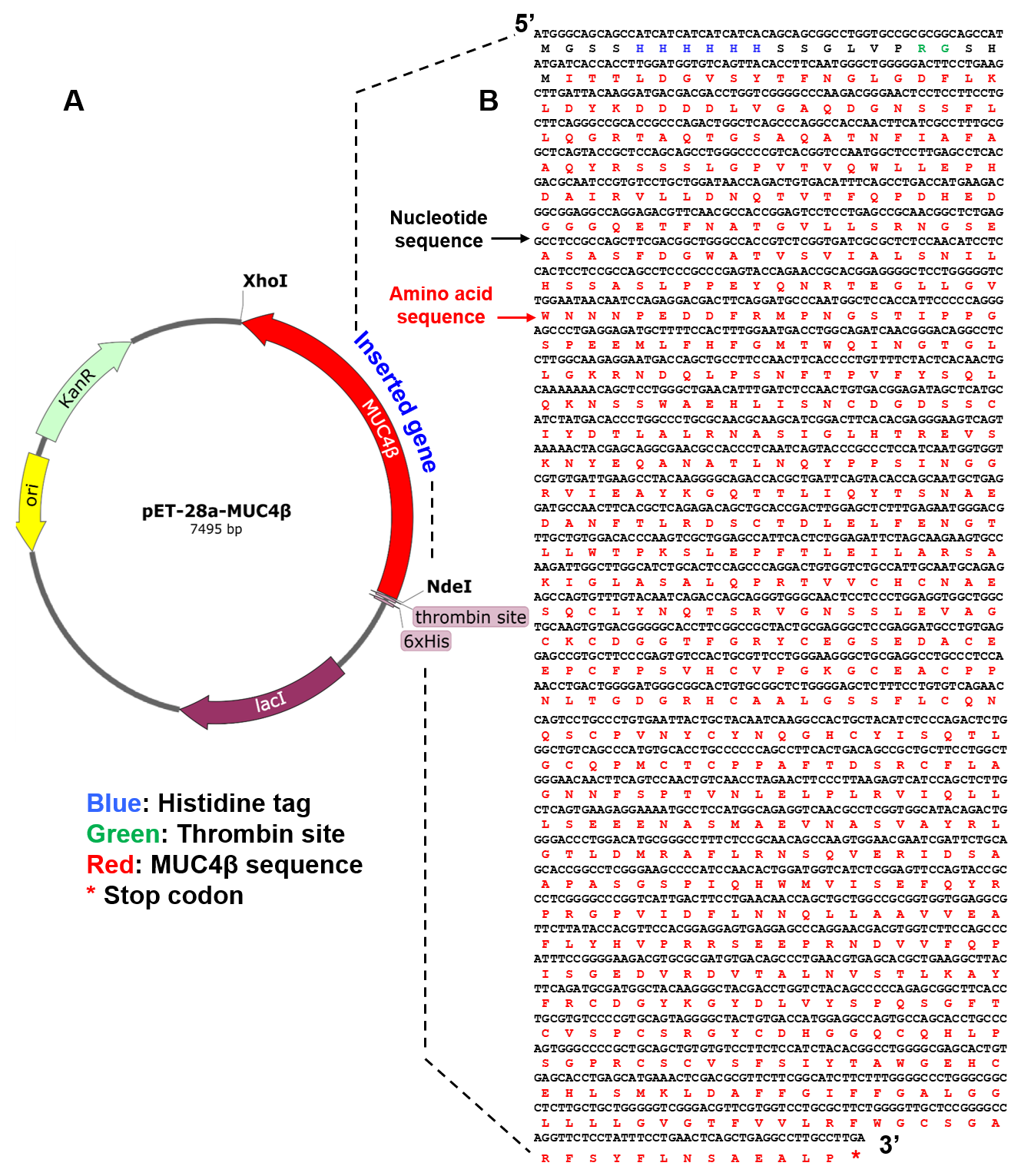


**Figure S1. Recombinant pET-28a-MUC4β vector construct (plasmid), nucleotide, and amino acid sequences of the inserted gene. (A)** Schematic diagram of the constructed pET-28a-MUC4β plasmid. **(B)** The nucleotide sequence (black) and amino acid sequence (red) encoding the *MUC4β* gene in-frame with the N-terminal His_6x_ tag (blue) and thrombin site (green). The plasmid map was generated using SnapGene Software (<http://www.snapgene.com> version 5).

**
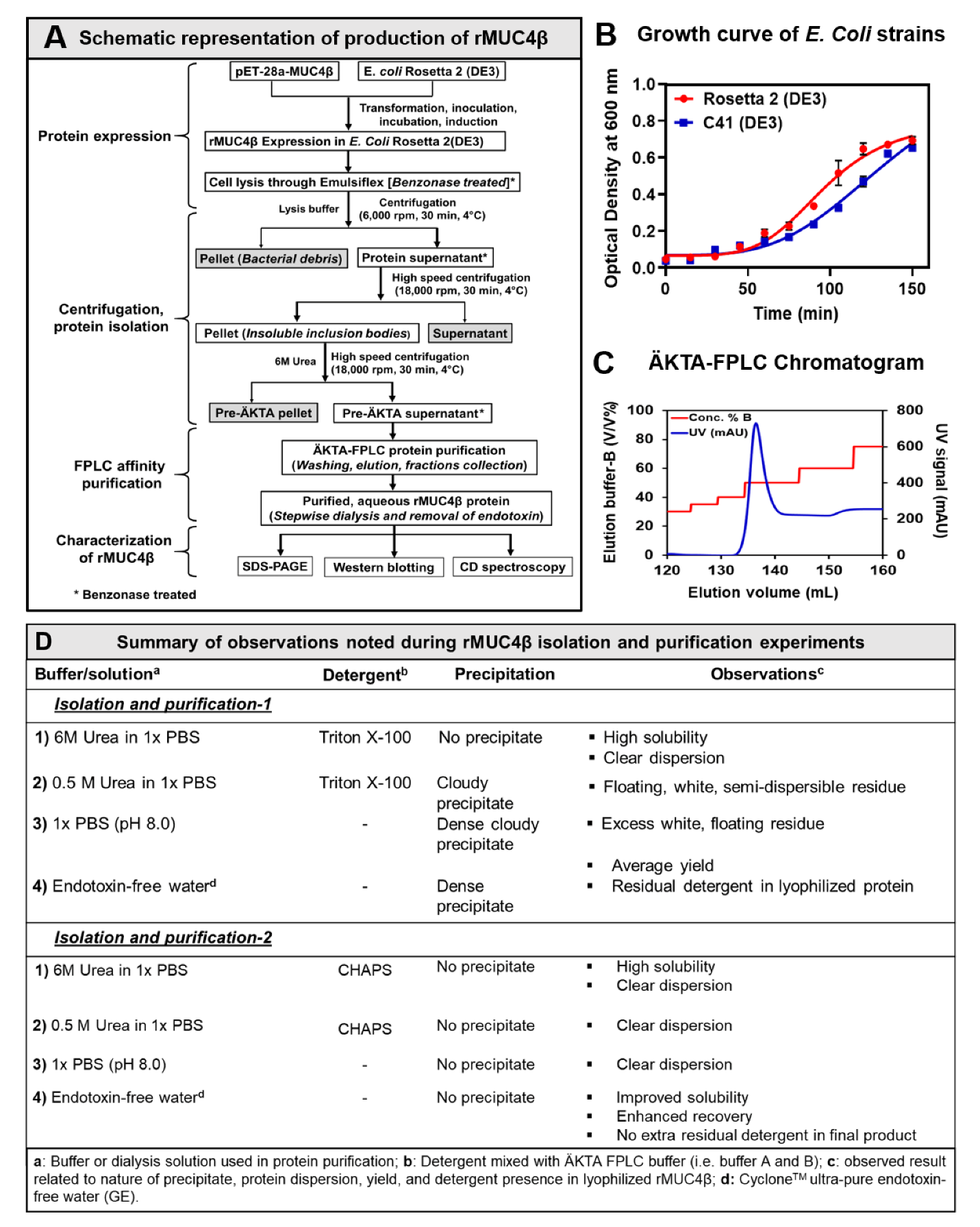
**

**Figure S2. rMUC4β protein isolation and ÄKTA-FPLC purification.** **(A)** Flow-chart is summarizing strategies for expression, isolation, purification, and characterization of rMUC4β. **(B)** Growth curve for pET-28a-MUC4β transformed *E. coli* strains: The cell growth plot shows the OD_600_ values of *E. coli* growth culture at variable time points. Each line signifies a sigmoidal fit of the corresponding growth point. The data points with the error bar represent mean ± SD. **(C)** Representative Ni-NTA affinity chromatogram: Chromatogram showing the step elution profile of rMUC4β recovered from Rosetta 2(DE3) cells using HisTrap Ni-NTA column on ÄKTA-FPLC system. A single, narrow peak (blue line, appeared at 130-140 mL elution) indicates the UV absorbance at 280 nm from the column eluate, which contained the desired protein. **(D)** Summary remark noted during rMUC4β isolation and purification experiments: Comparative observations made during the head-to-head assessment of two detergents based on protein dispersion, status of its precipitation, and yield. The flow diagram was S2A, and S2D was designed by using Microsoft Powerpoint (<https://www.microsoft.com/en-us/microsoft-365/powerpoint>). Figure S2B (growth curve) and Figure S2C (chromatogram) were plotted by using Microsoft Excel (<https://www.microsoft.com/en-us/microsoft-365/excel>)

**
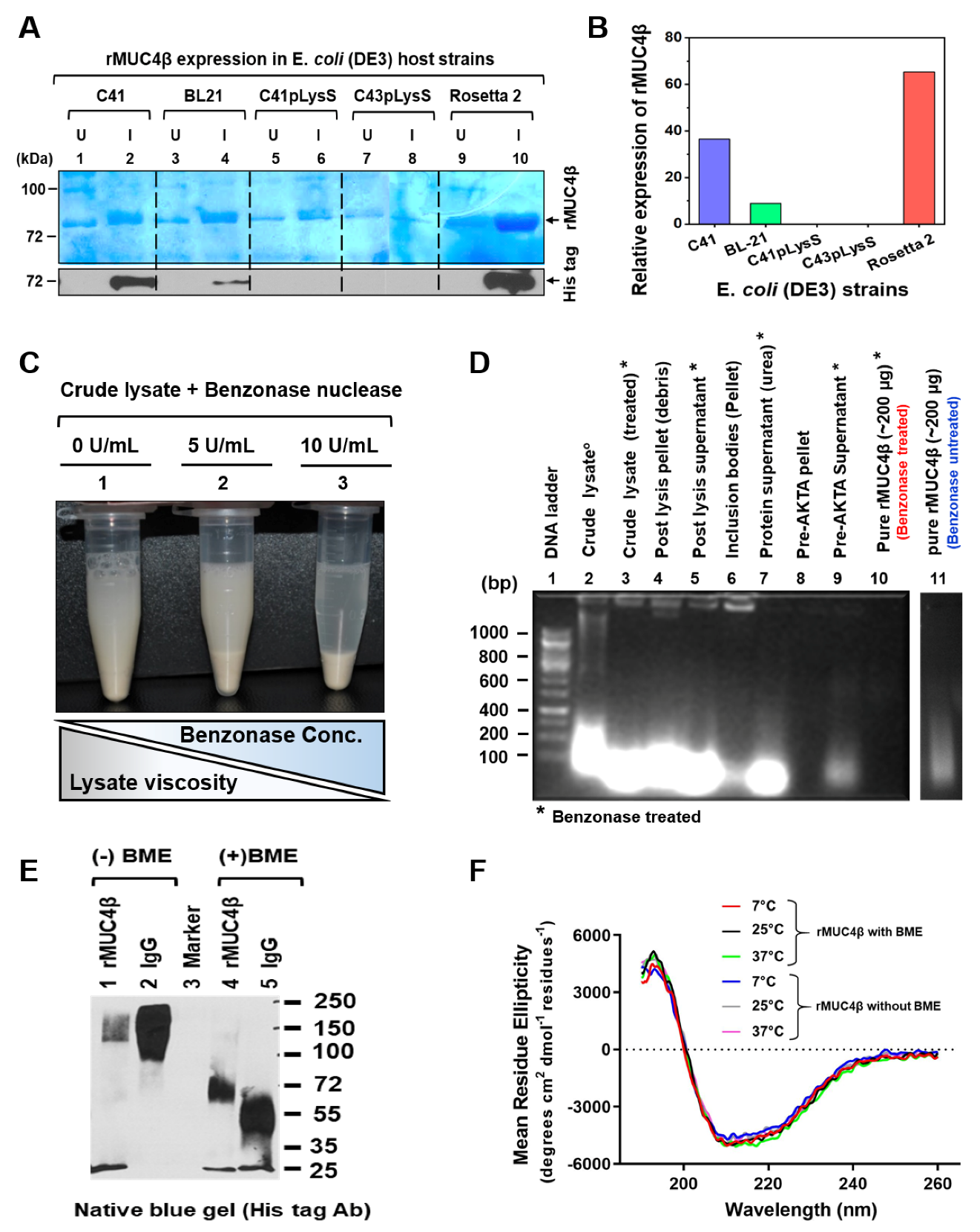
**

**Figure S3. Optimization of conditions for rMUC4β expression. (A)** Effect of different *E. coli* (DE3) strains on rMUC4β expression efficiency: SDS-PAGE and immunoblotting analyses were performed on the cultures of various strains prior to (U) or following (I) induction with IPTG. **(B)** The bar graph indicating relative protein expression levels from different *E. coli* strains. Band signal intensity from the immunoblot was analyzed by Image J (<https://imagej.nih.gov/ij/>). **(C)** Effect of Benzonase nuclease on the cell lysate viscosity. Aliquots of MUC4β-expressing Rosetta 2 (DE3) lysates [0.5 g/mL of lysis buffer] were treated with the indicated amount of Benzonase at 25°C for 10 min centrifuged at 1000 rpm for 3 min and photographed using Nikon DSLR. **(D)** Agarose gel electrophoretic analysis of nucleic acid content in various steps during affinity purification. The untreated *E. coli* cell lysates (lane 2) benzonase-treated clarified fractions (lanes 3, 5, 7, 9), purified rMUC4β (~ 200 μg each) recovered from cell lysate with (red, lane 10) and without (blue, lane 11) benzonase treatment. **€** Representative western blot analysis of purified rMUC4β protein following Blue Native polyacrylamide gel electrophoresis (BN-PAGE): The rMUC4β protein (+/- BME) and IgG antibody (+/- BME) were subjected to the BN-PAGE, resolved, transferred, and probed with the anti-His tag mouse antibody (clone 27E8). **(F)** Far-UV CD spectra of rMUC4β protein with and without the treatment of BME and at 7°C, 25 °C, and 37 °C. The Figure S3B and S3F graphs were plotted using GraphPad Prism (<https://www.graphpad.com/> version 8).

**
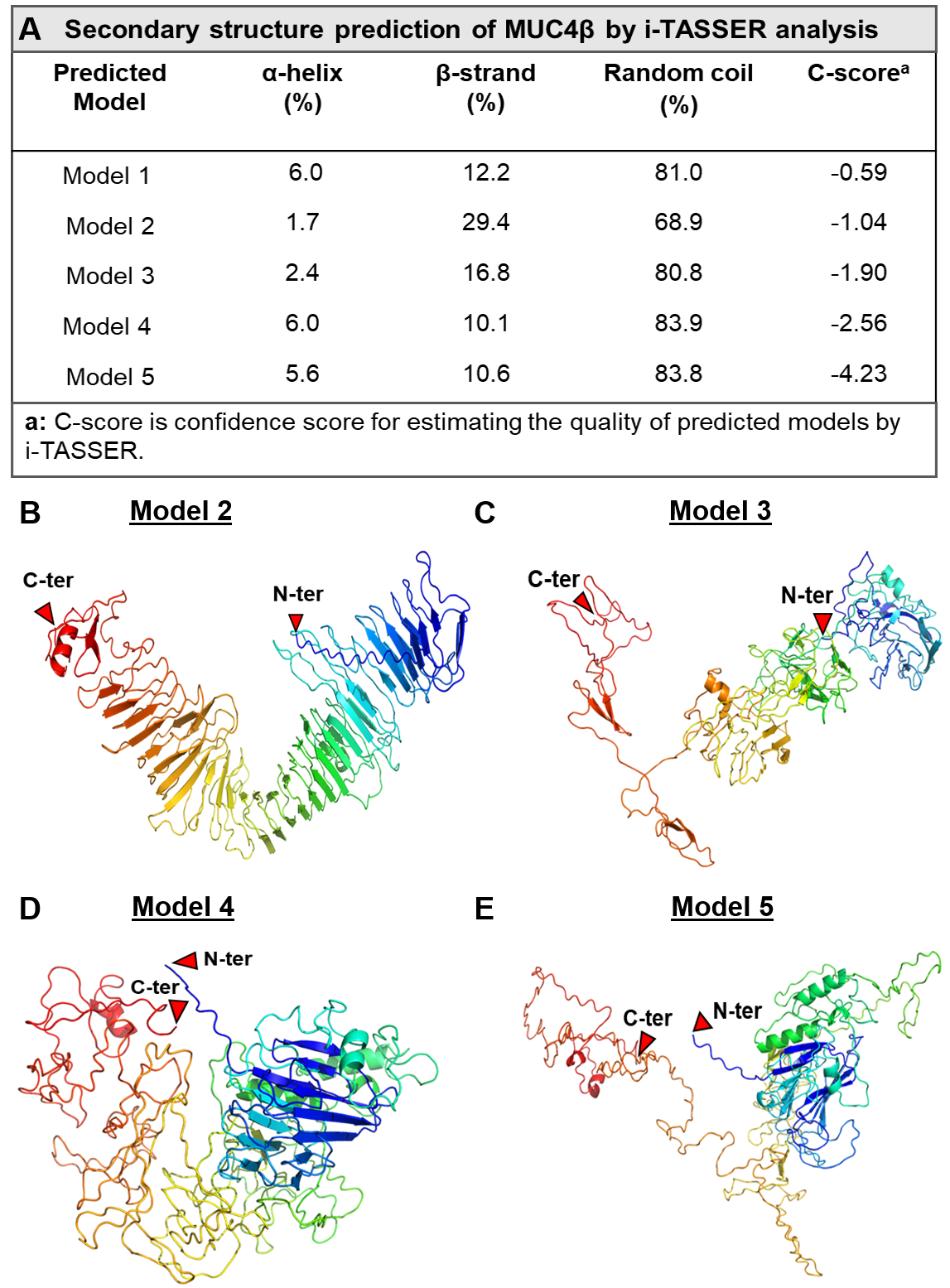
**

**Figure S4. Secondary structure prediction of rMUC4β by I-TASSER. (A)** Table showing the secondary structure predicted by i-TASSER (<https://zhanggroup.org/I-TASSER/>). The five models generated by the server are arranged based on their C-score (confidence score). Model 1 is shown in Figure 2E, and **(B, C, D, and E)** are the other four predicted models of rMUC4β (colored in the rainbow spectrum) generated with the I-TASSER tool. The protein structure was generated using PyMOL Molecular Graphics System, Schrödinger, LLC (<https://pymol.org/2/> version 2.3.4).

**
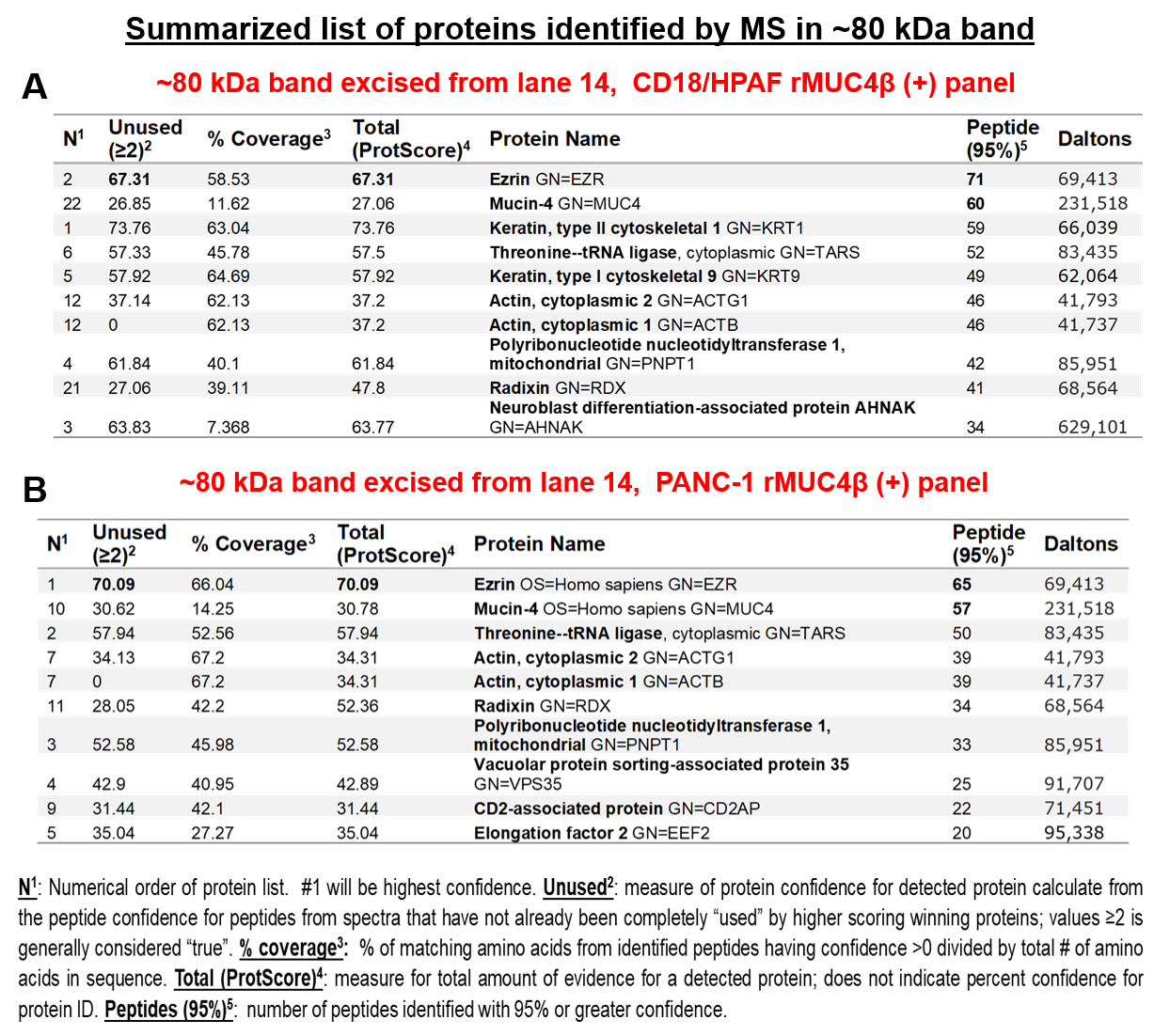
**

**Figure S5. List of proteins identified through mass spectrometry in the ~80 kDa band** **and proteomic analysis performed using Mascot**. The unique bands positioned at ~80 kDa (Figure 3B/3C, rMUC4β panel, lanes 13, arrow marked band) were excised from CD18/HPAF. **(A)** and PANC-1. **(B)** pull-down reactions and submitted for MS analysis. The proteins are listed in the order of the number of peptides showing >95% confidence. A legend describing the terms denoted in the A and B is shown below the table.

**Supplementary Figure S6-S17**

Following supplementary figures (S6-S17) include the full-length SDS-PAGE gel or western blot (Black box) and the cropped sections (Red dotted box). All western blot membranes are developed by the Konica X-ray film processor (Model: SRX-101A) except Figure 4 (lower panel, MUC4-6E8 Ab and β-actin blots), which was imaged by using the ibright imaging system (Model: ibright 1500, Invitrogen [Thermo Fisher Scientific]).

**
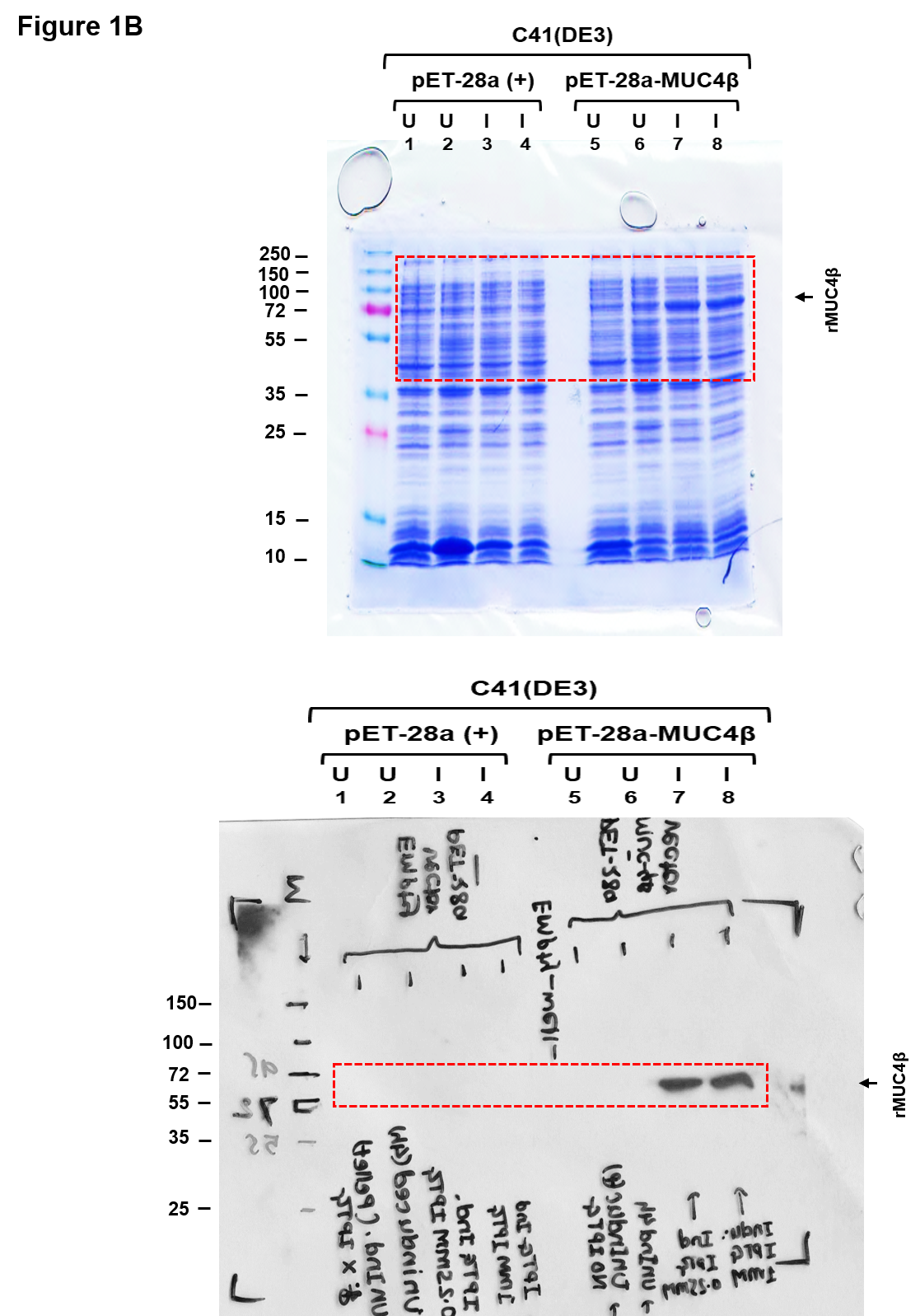
**

**Figure S6.** Expression profile of total cellular proteins from E. coli C41(DE3) strain **(Figure 1B).**

**
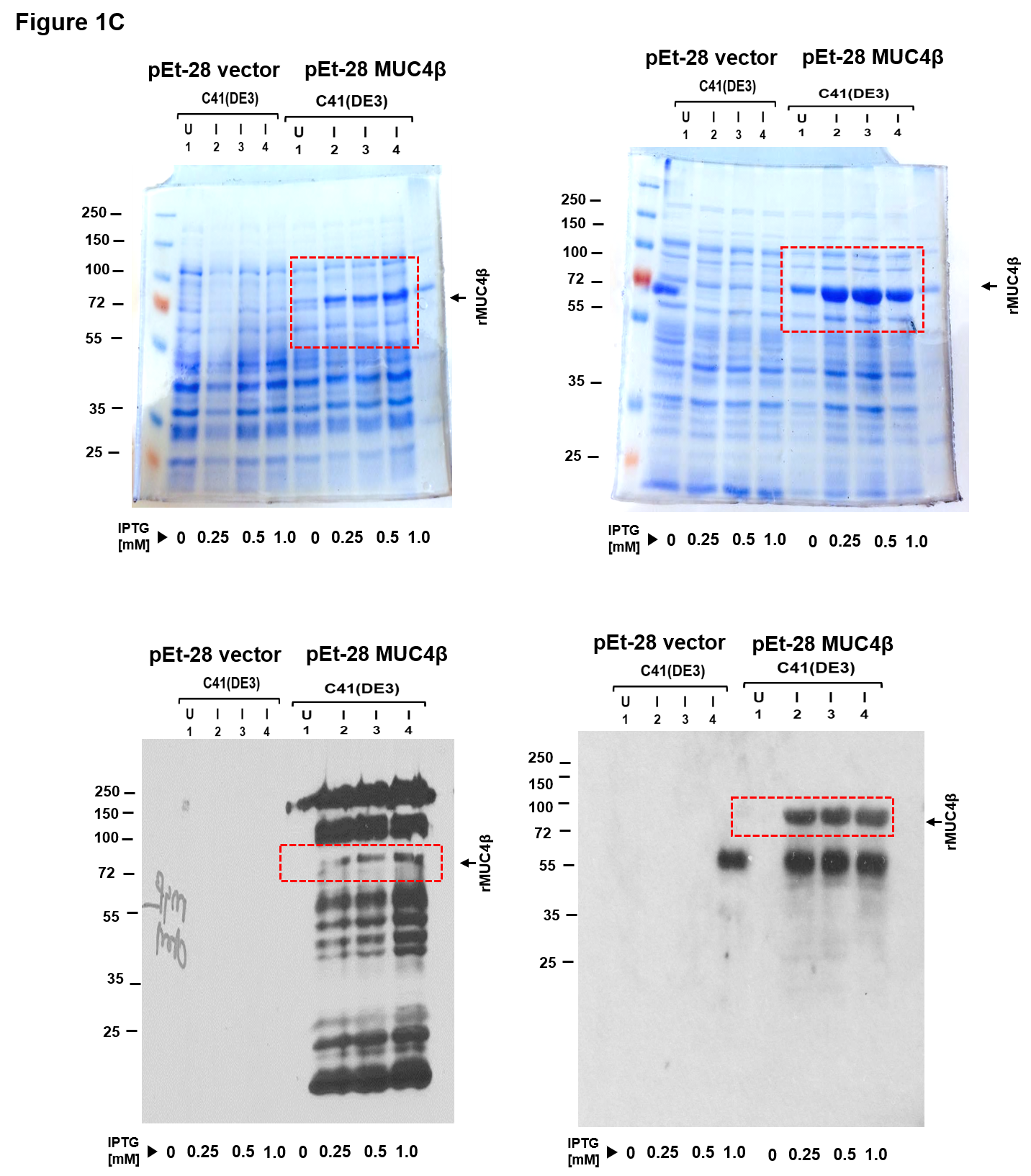
**

**Figure S7.** Comparative assessment of rMUC4β expression profile in C41(DE3) and Rosetta 2(DE3) competent cells at different IPTG concentrations **(Figure 1C).**

**
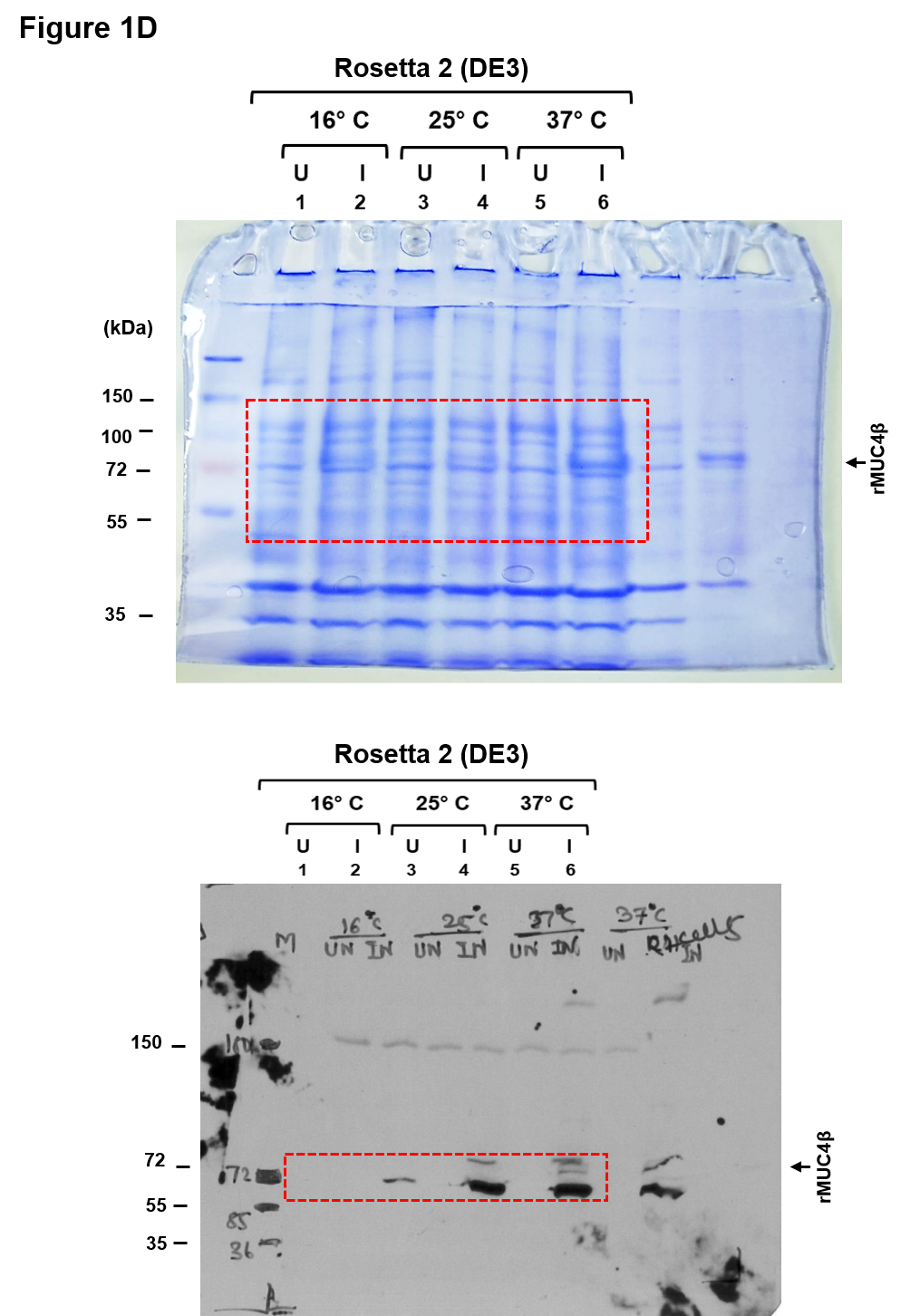
**

**Figure S8.** Effect of different post-induction incubation temperatures on rMUC4β expression in Rosetta 2(DE3) **(Figure 1D).**


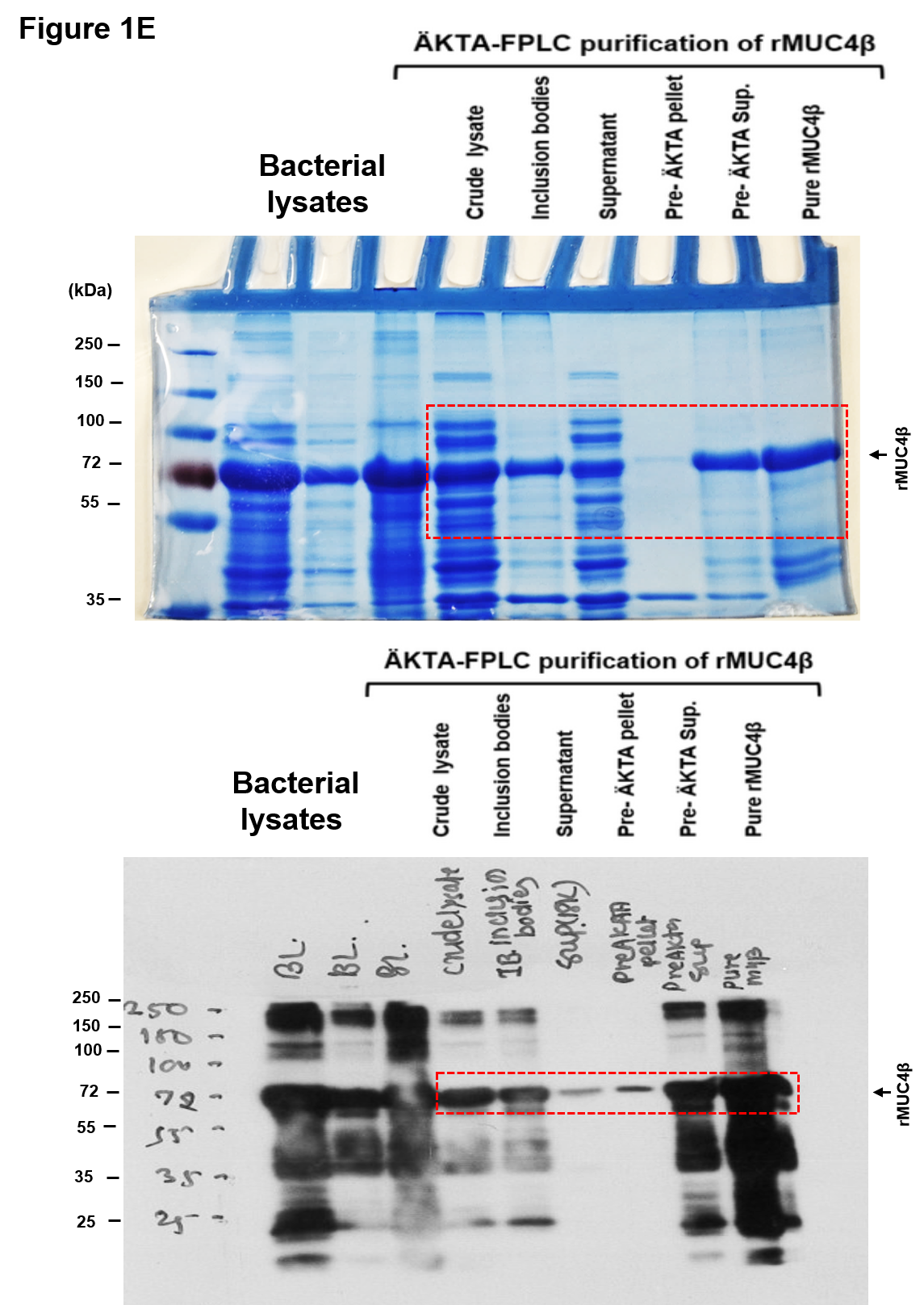


**Figure S9.** Isolation and ÄKTA-FPLC affinity purification of rMUC4β from crude lysate **(Figure 1E)**.

**
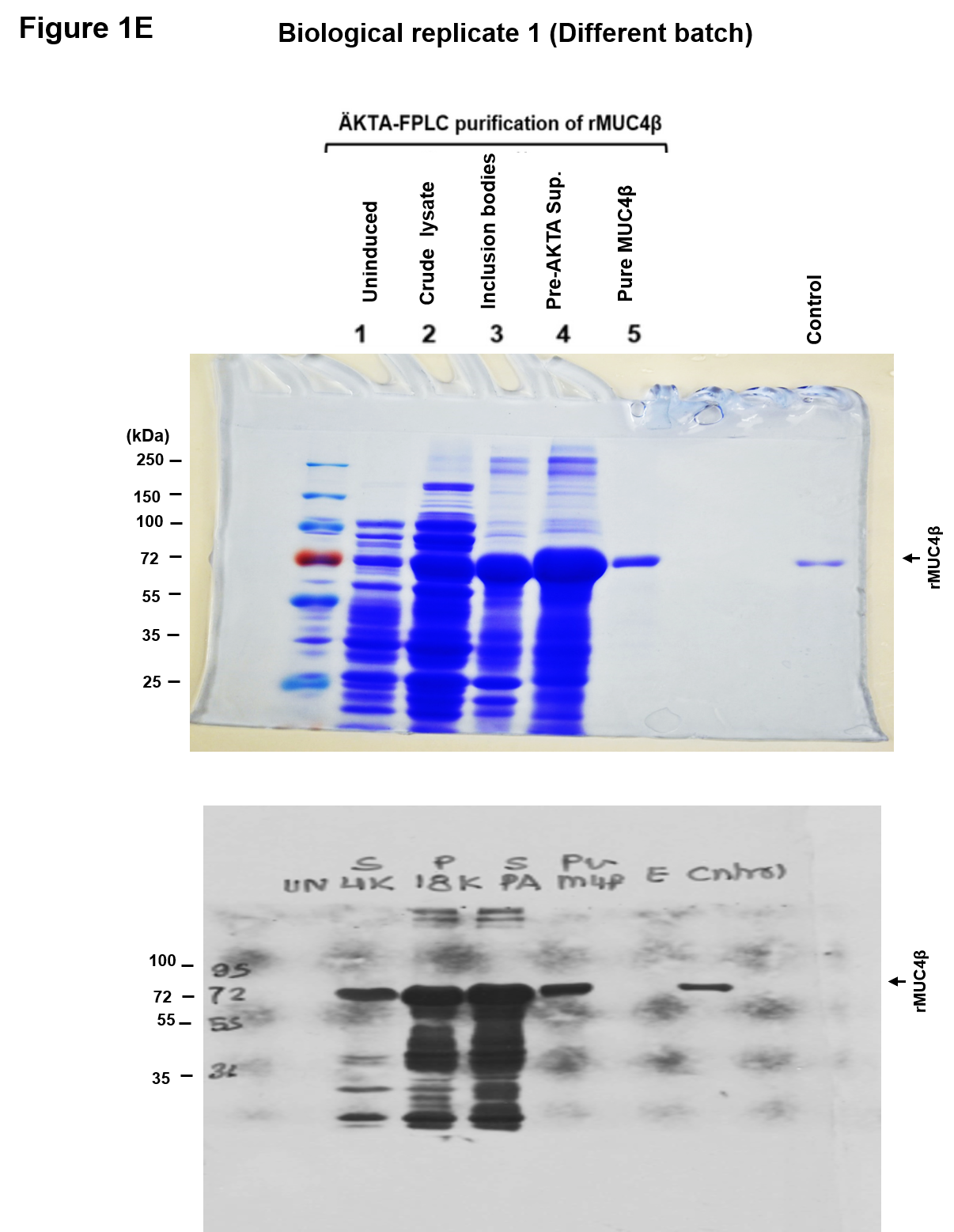
**

**Figure S10.** Isolation and ÄKTA-FPLC affinity purification of rMUC4β from crude lysate **(Figure 1E, Biological replicate 1)**.

**
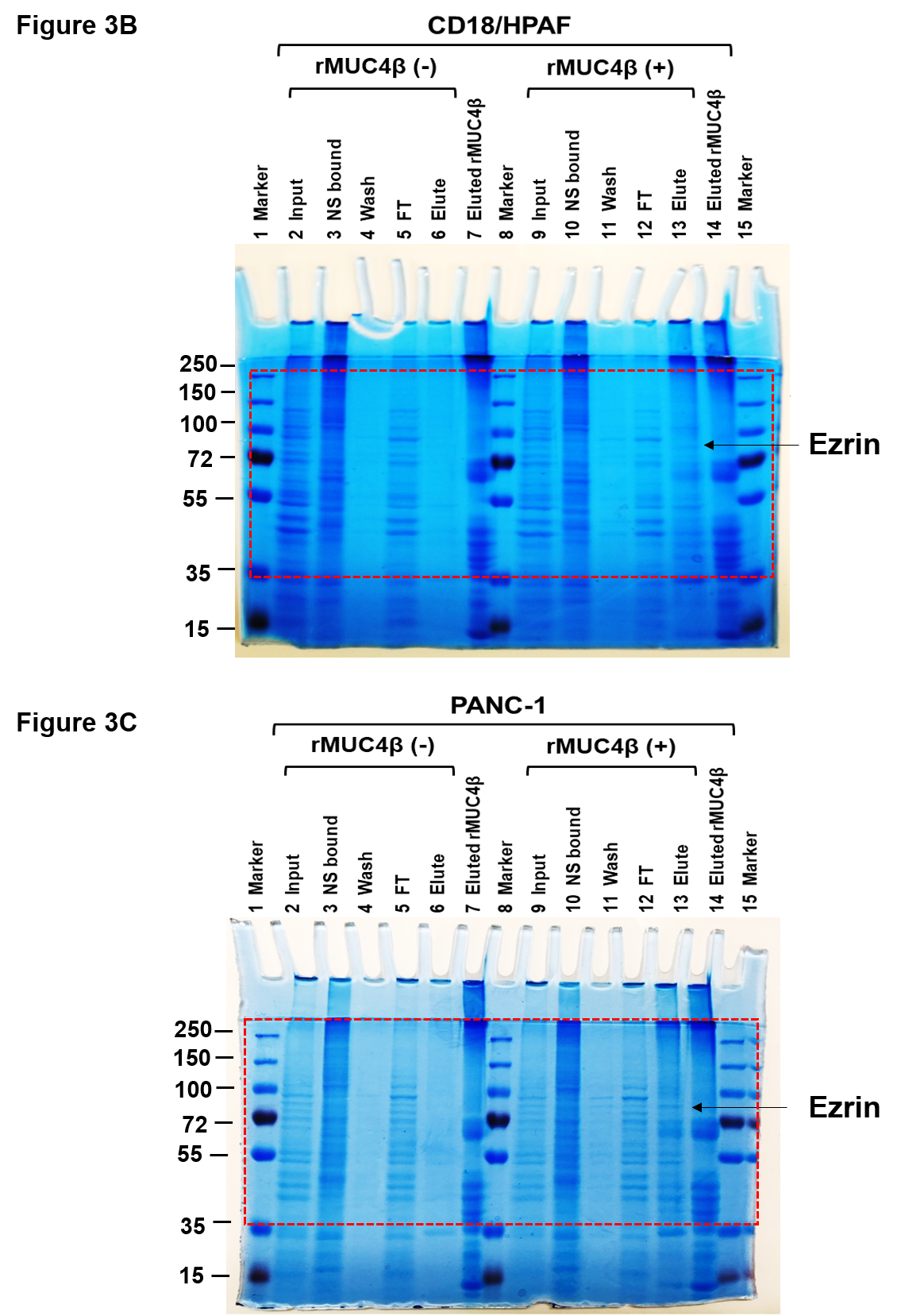
**

**Figure S11.** Expression profile of saved fractions from the pull-down assay examined by the SDS-PAGE gel and stained with Coomassie Blue **(Figure 3B, 3C)**.

**
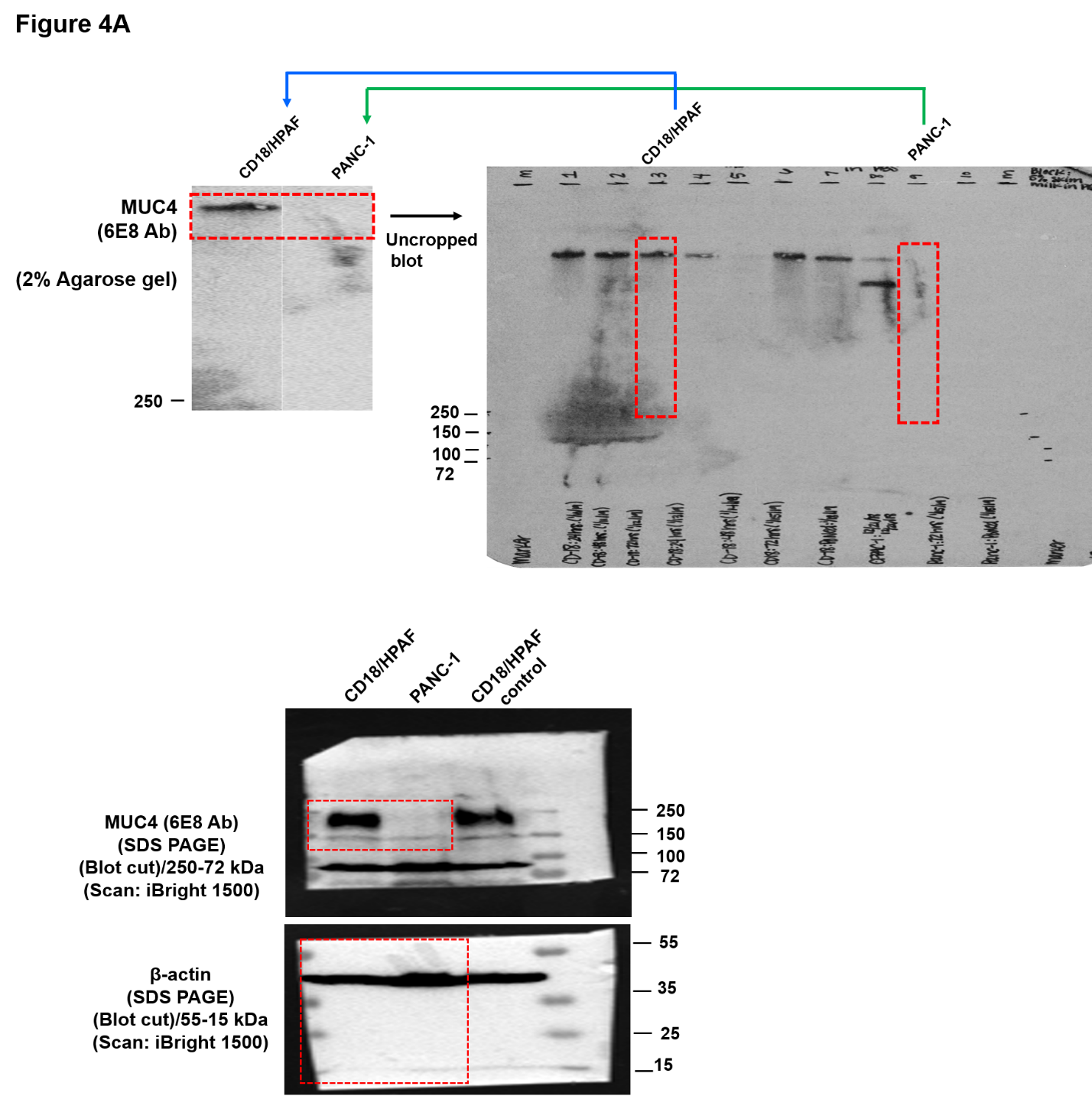
**

**Figure S12.** Expression profile of MUC4 in CD18/HPAF and PANC-1 cells determined by immunoblotting following 2% agarose gel electrophoresis (upper panel) and 10% SDS-PAGE gel (lower panel). The blots were imaged using the ibright imaging system (Model: ibright 1500, Invitrogen [Thermo Fisher Scientific]) **(Figure 4A)**.

**
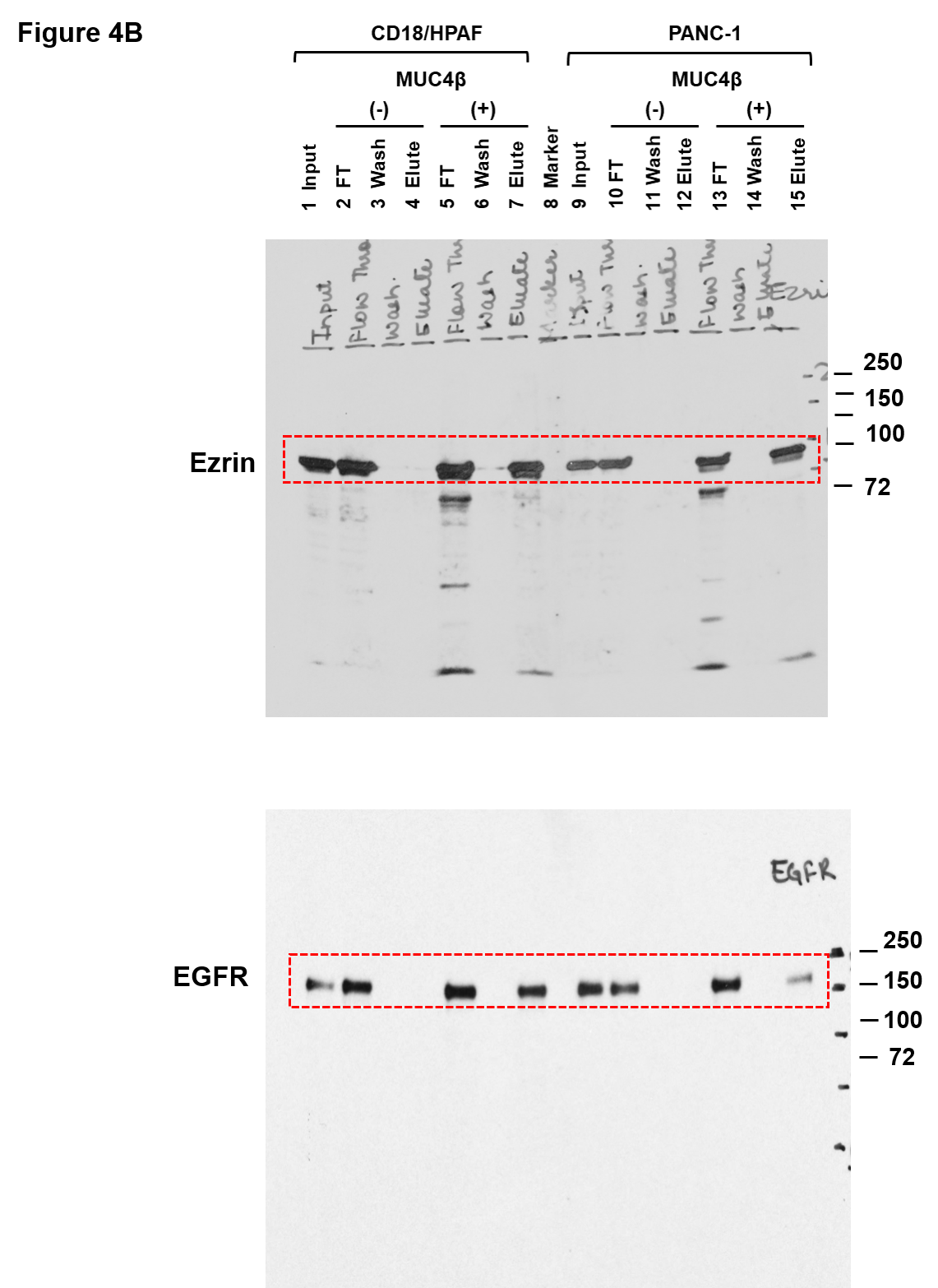

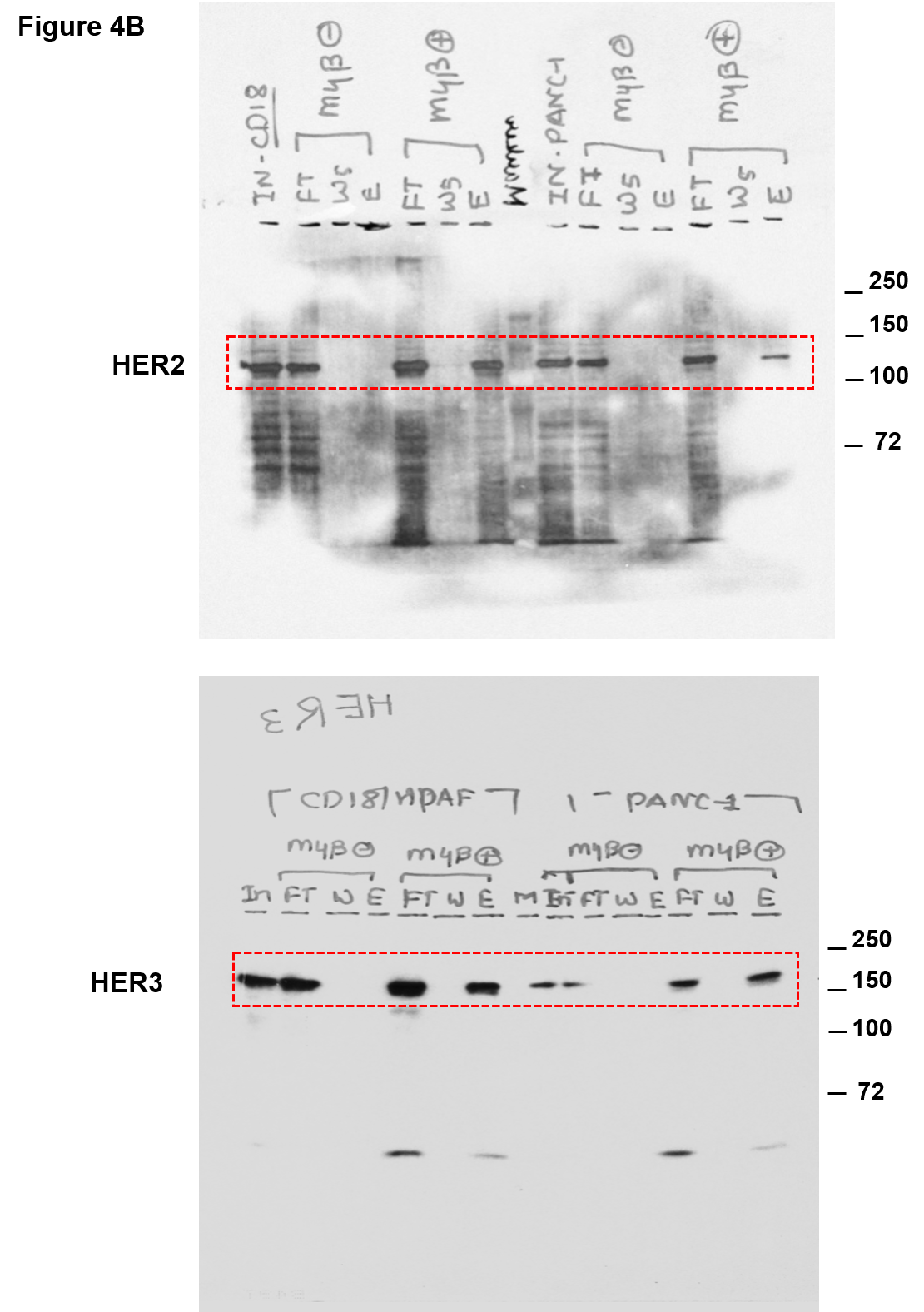
**

**
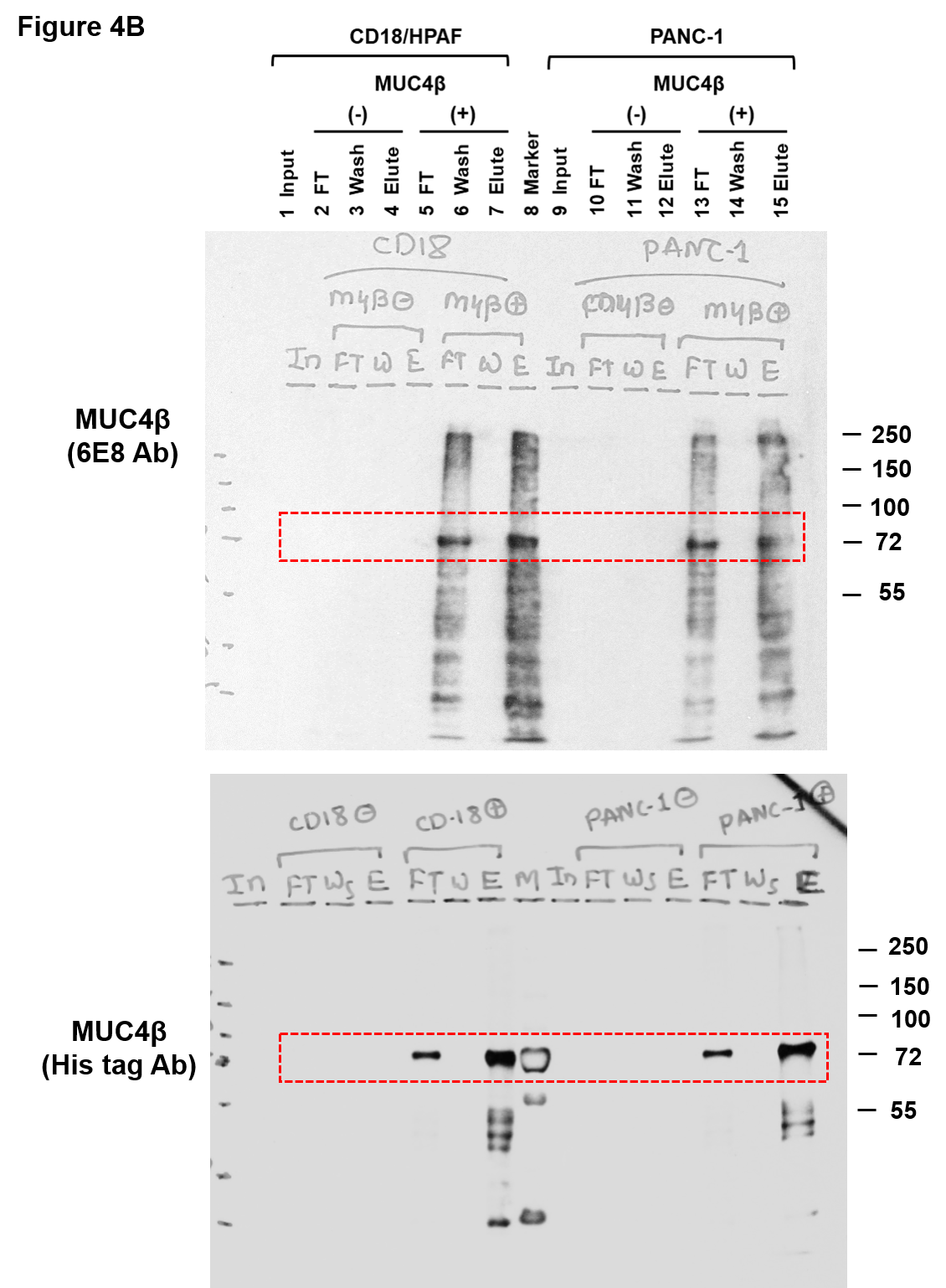
**

**Figure S13.** Immunoblot analyses of Ni-Ni-NTA pull-down fractions from CD18/HPAF or PANC-1 cell lysates in the presence (+) or absence (-) of rMUC4β. The blots were probed with the indicated antibodies **(Figure 4B).**

**
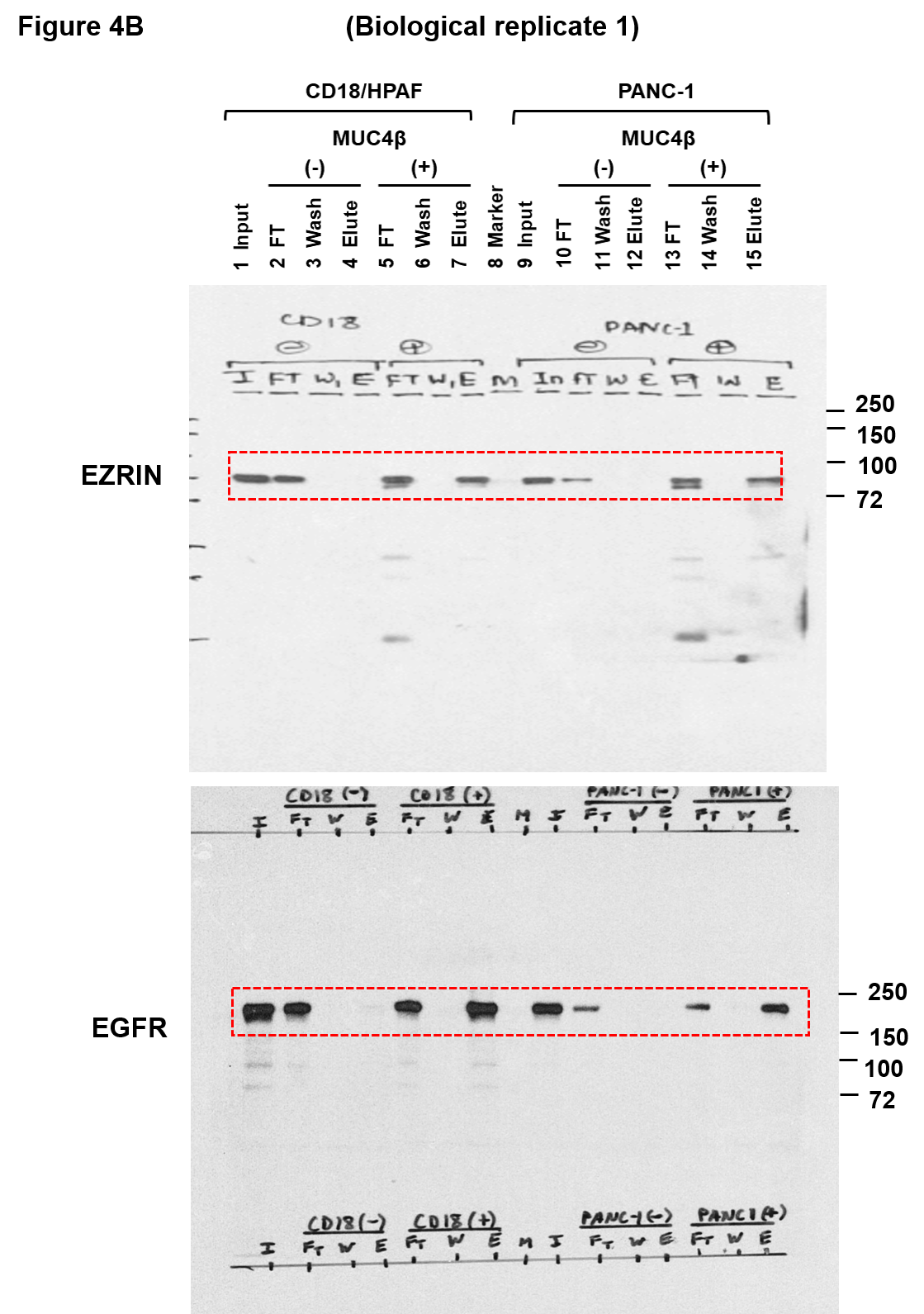
**

**
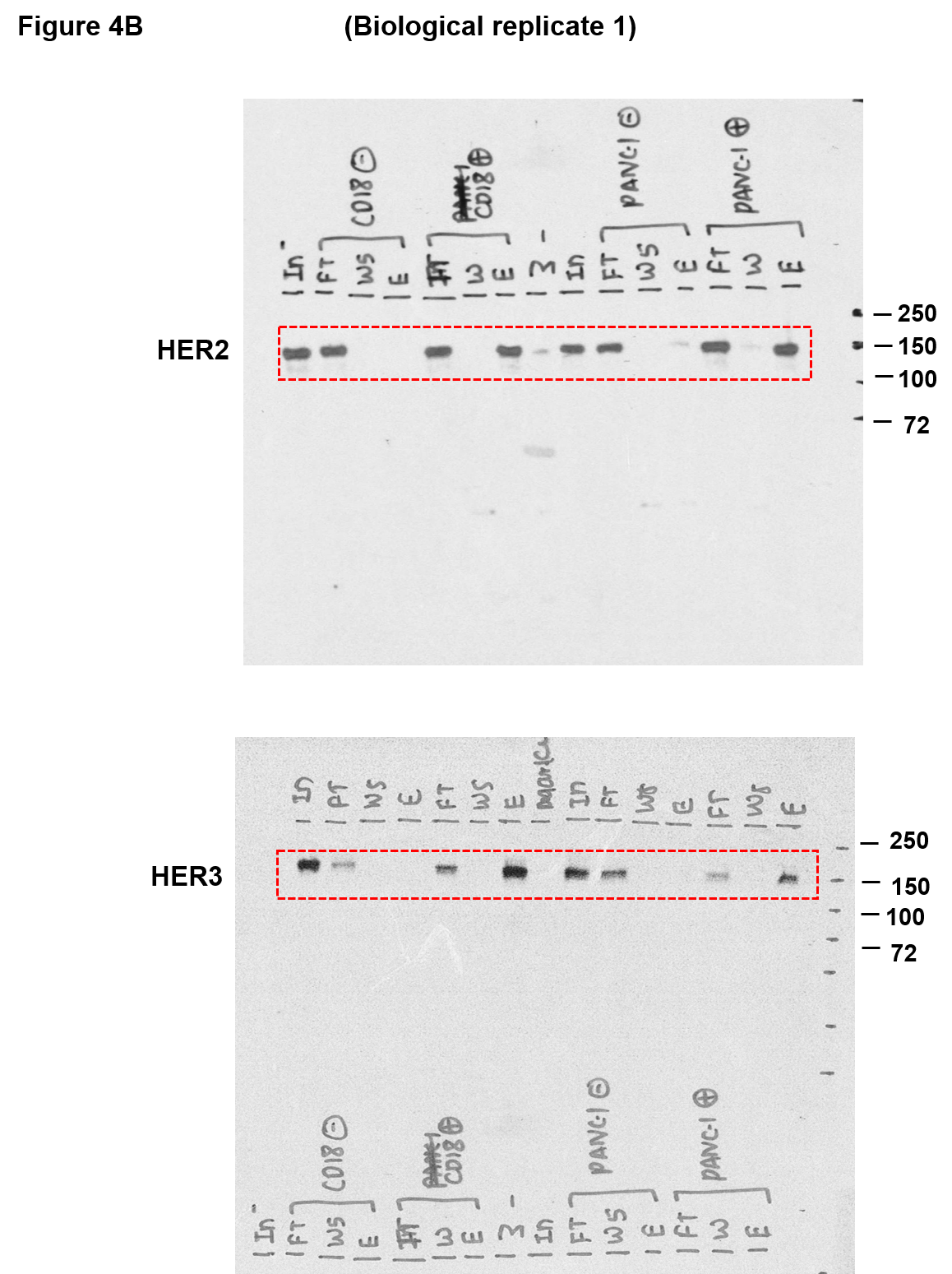
**

**
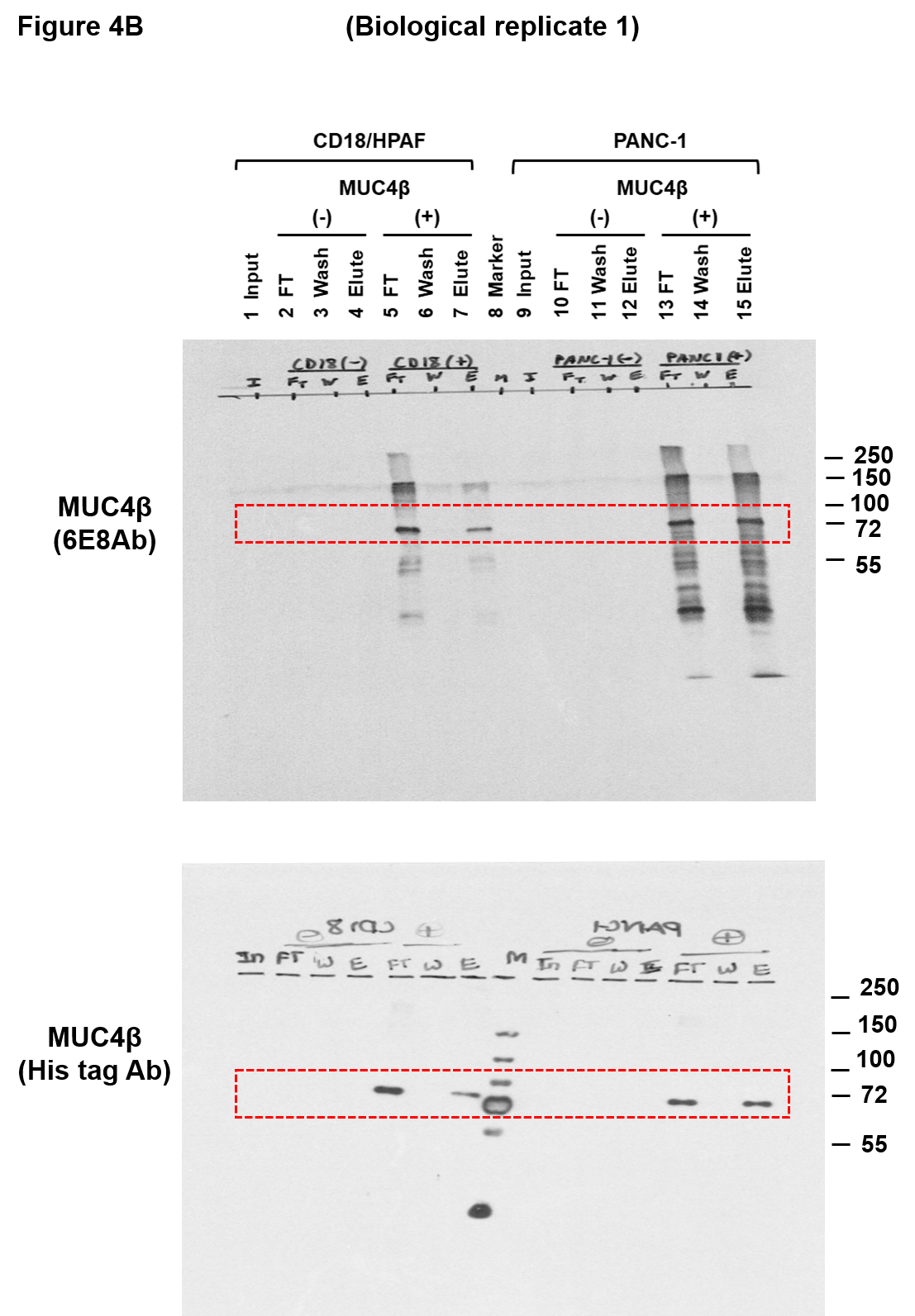
**

**Figure S14.** Immunoblot analyses of Ni-Ni-NTA pull-down fractions from CD18/HPAF or PANC-1 cell lysates in the presence (+) or absence (-) of rMUC4β. The blots were probed with the indicated antibodies**.** The replicate of the pulldown experiment was performed independently by one of the co-authors **(Figure 4B, Biological replicate 1)**.

**
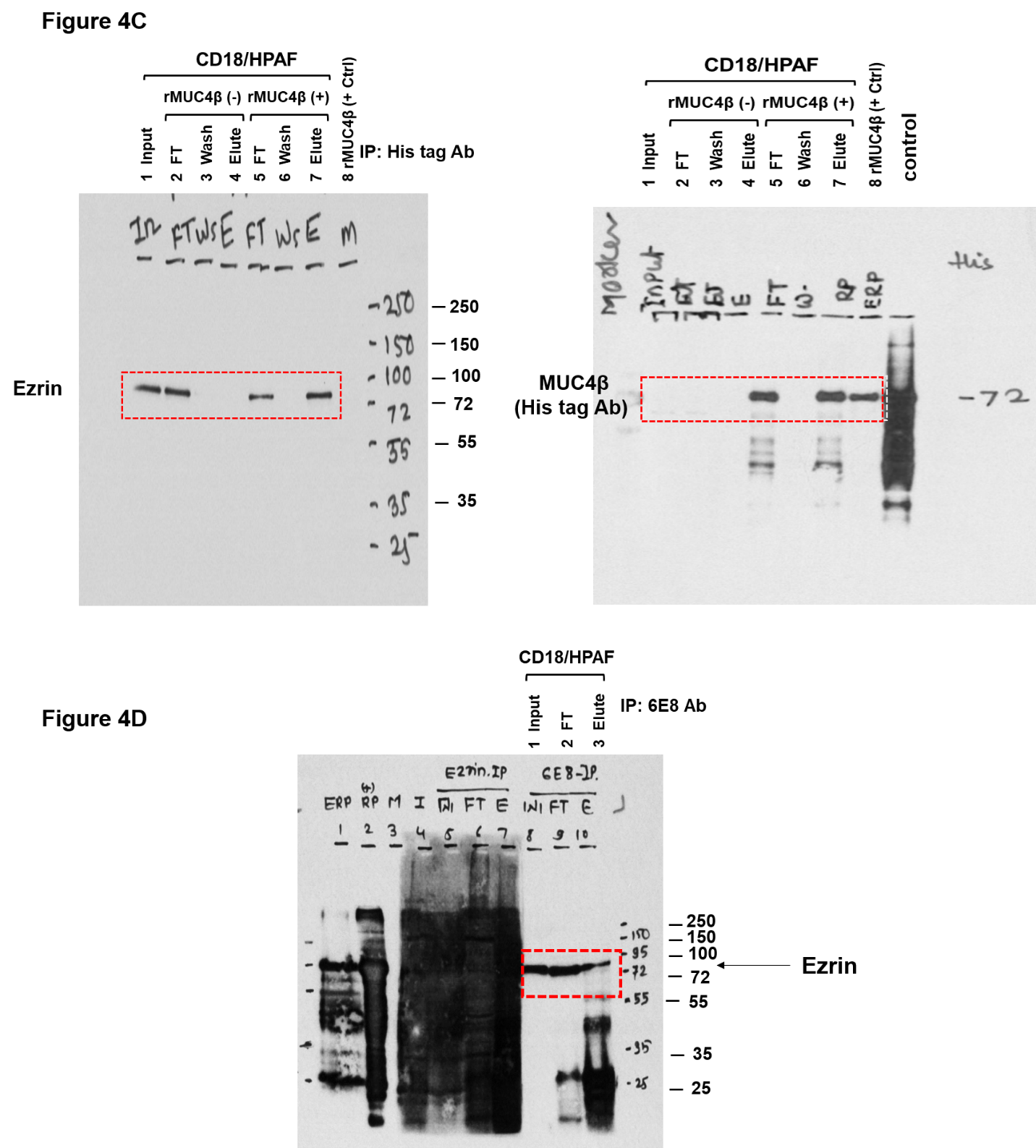
**

**Figure S15. Upper panel:** Immunoprecipitation assay performed using an anti-His tag Ab (clone 27E8) conjugated to magnetic beads following incubation of CD18/HPAF cell lysate in the presence and absence of rMUC4β. The membranes were probed with Ezrin (left blot) and His tag (right blot) antibodies (**Figure 4C). Lower panel:** Immunoprecipitation assay to study the interaction of endogenous MUC4 after mixing the protein A/G agarose beads and CD18/HPAF lysates following incubation with anti-MUC4β Ab **(Figure 4D)**.

**
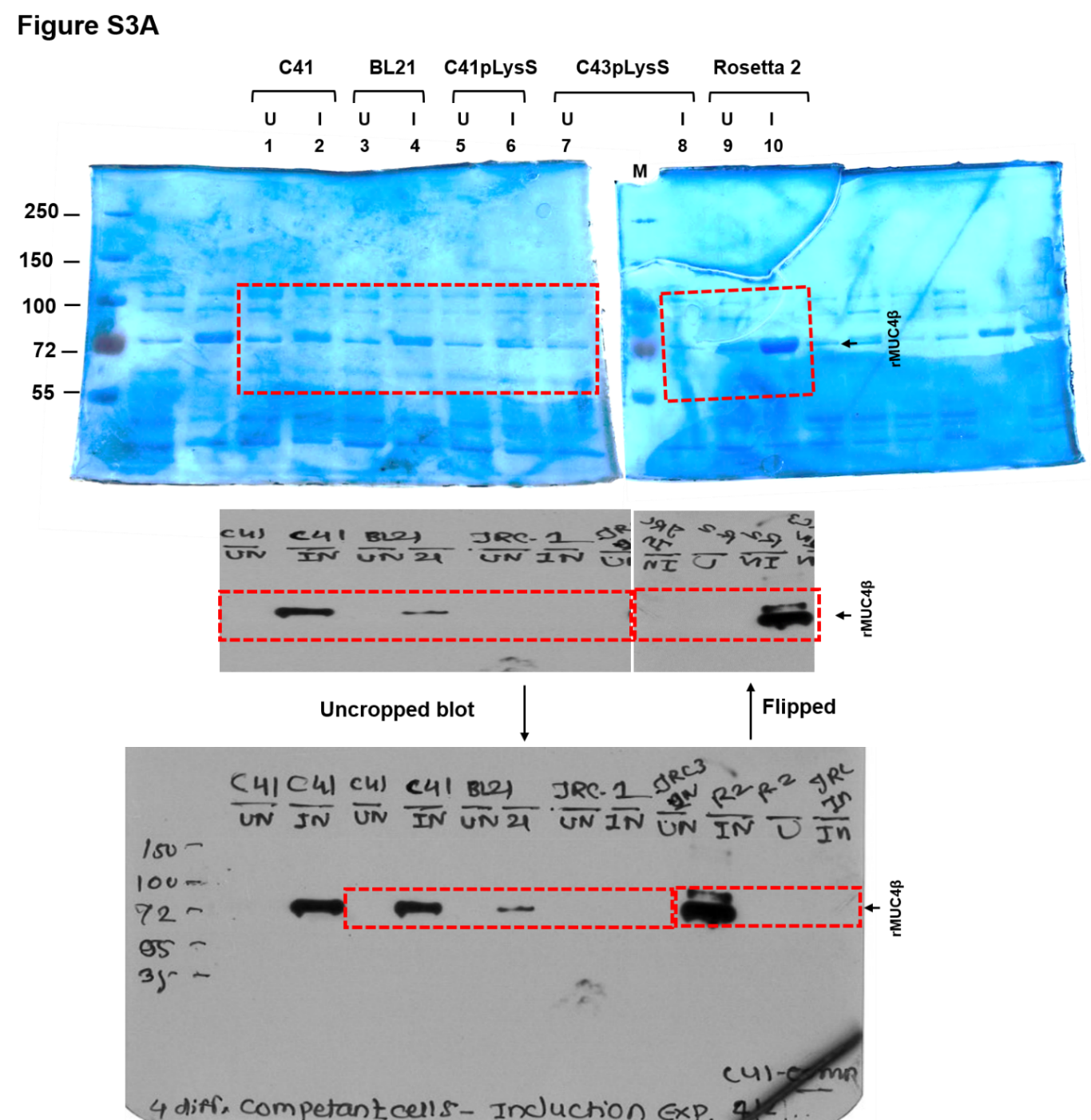
**

**Figure S16.** Effect of different *E. coli* (DE3) strains on rMUC4β expression efficiency **(Figure S3A)**.

**
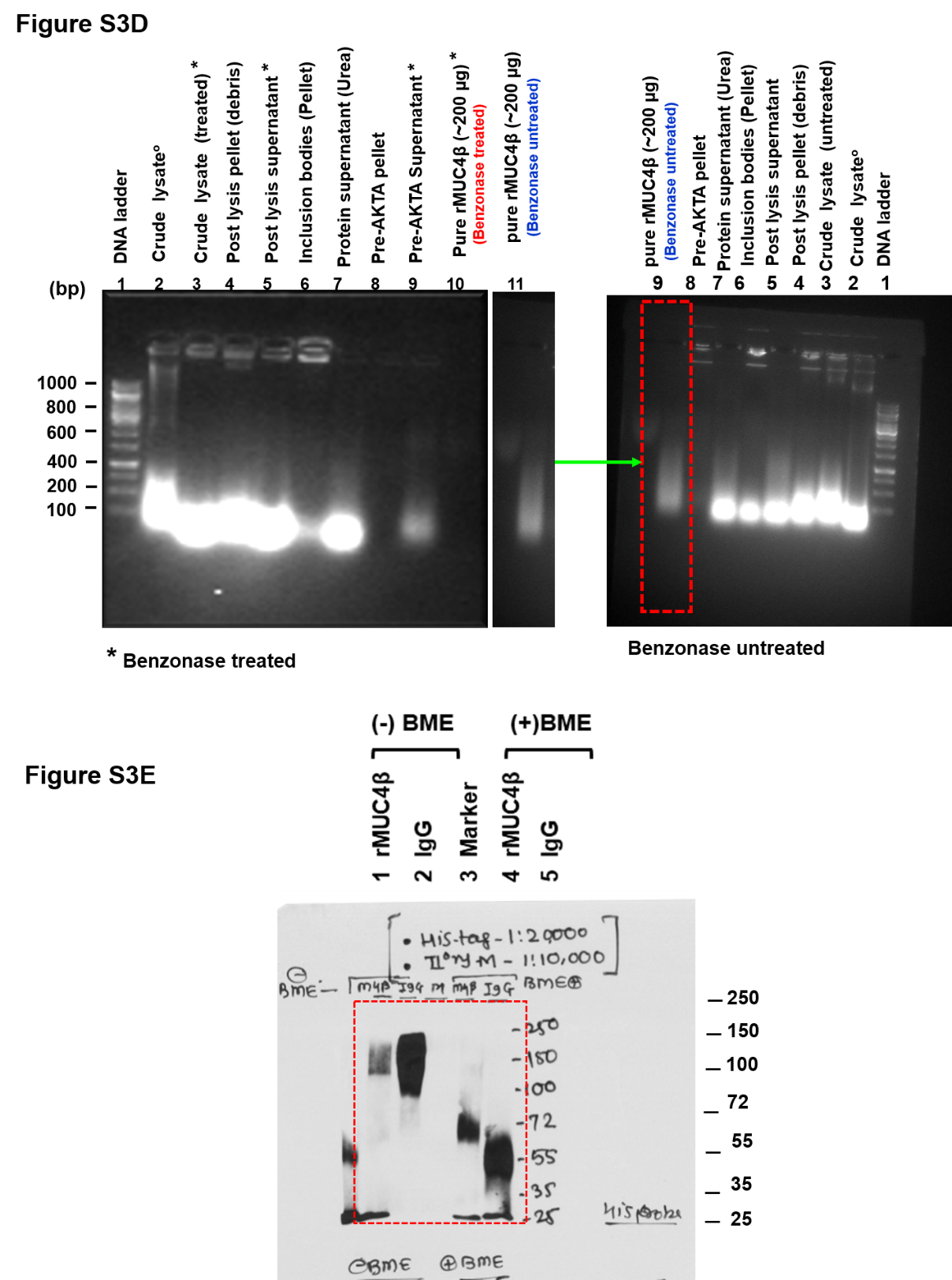
**

**Figure S17. Upper panel:** Agarose gel electrophoretic analysis of nucleic acid content in various steps during affinity purification. Lane 11 is without benzonase treatment. **(Figure S3D)**. **Lower panel:** Representative western blot analysis of purified rMUC4β protein following Blue Native polyacrylamide gel electrophoresis (BN-PAGE) **(Figure S3E)**.

**Supporting methods**

**S1. Construction of recombinant plasmid encoding for MUC4β expression**

MUC4β sequence was amplified from the mini-MUC4 gene^1^ using two PCR primers; the forward primer: (5’-GTTATCATATGATCACCACCTTGGATGGTGTCAG), and reverse primer: (CTTATCTCGAGTCAAGGCAAGGCCTCAGC-3’). Sequence amplification was completed in the Expand Long Template PCR system (Roche). The obtained PCR product containing 2199 bp MUC4β sequence and upstream His_6x_ tag and cleavable thrombin site was ligated into the NdeI and XhoI sites of pET-28a (+) vector (Novagen). The resulting cloned sequence had an N-terminal hexahistidine tag and was verified by sequencing.

**S2. Cell culture and protein lysate preparation**

Human pancreatic cancer cell lines, CD18/HPAF (endogenous MUC4 expressing) and PANC-1 (MUC4 non-expressing) ^2^, were cultured in DMEM supplemented with 10% heat-inactivated fetal bovine serum and penicillin/streptomycin (100 μg/mL) at 37°C and 5% CO_2_. Cells were passaged at ~80% confluency using 0.25% (w/v) trypsin solution containing 0.04% (w/v) EDTA. Total cell lysates were made on ice by scraping cells into RIPA buffer (10 mM Tris-HCl pH 8.0, containing 150 mM NaCl, 2mM phenylmethylsulfonyl fluoride, 10 mM NaF, 1 mM sodium orthovanadate (Na_3_VO_4_) and protease inhibitor cocktail).

**S3. SDS-PAGE and immunoblot analysis**

Samples were resolved on 10 or 12% SDS-PAGE gels and stained with Coomassie blue to visualize and track the purity of rMUC4β at various stages of the purification process. For western blotting, electrophoretically resolved proteins were transferred to polyvinylidene difluoride (PVDF) membranes, blocked with either 1% BSA-TBST (1ᵡ Tris-buffered saline and 0.1% Tween 20) or 5% non-fat dry milk in PBST (1ᵡ phosphate buffer saline (PBS) and 0.1% Tween 20) and incubated with anti-Ezrin (1:3000), total EGFR (1:1000), anti-HER2 (1:1000), and anti-HER3 (1:1000) diluted in 1% BSA/TBST. Anti-His tag Ab (1:30,000) and lab-made anti-MUC4β Ab (Clone 6E8, 0.02 μg/mL) were diluted in 5% non-fat dry milk in PBST. Membranes were then washed extensively in TBST and incubated with HRP-conjugated goat anti-mouse IgG secondary Abs for 60 min. After washing extensively with TBST, a signal was developed using ECL chemiluminescence (Thermo Scientific).

**S4. References**

1. Moniaux, N. et al. Human MUC4 mucin induces ultra-structural changes and tumorigenicity in pancreatic cancer cells. *British journal of cancer* **97**, 345-357 (2007).

2. Kumar, S. et al. NCOA3-mediated upregulation of mucin expression via transcriptional and post-translational changes during the development of pancreatic cancer. *Oncogene* **34**, 4879-4889 (2015).
